# Antimicrobial Smart Dressings for Combating Antibiotic Resistance in Wound Care

**DOI:** 10.3390/ph18060825

**Published:** 2025-05-30

**Authors:** Alina-Georgiana Cristea (Hohotă), Elena-Lăcrămioara Lisă, Simona Iacob (Ciobotaru), Ionut Dragostin, Claudia Simona Ștefan, Iuliu Fulga, Andra Monica Anghel (Ștefan), Maria Dragan, Ionela Daniela Morariu, Oana-Maria Dragostin

**Affiliations:** 1 Research Centre in the Medical-Pharmaceutical Field, Faculty of Medicine and Pharmacy, “Dunarea de Jos” University, 800201 Galati, Romaniaionut.dragostin@yahoo.com (I.D.); claudia.stefan@ugal.ro (C.S.Ș.); fulgaiuliu@yahoo.com (I.F.);; 2 Department of Pharmaceutical Sciences II, Faculty of Pharmacy, “Grigore T. Popa” University of Medicine and Pharmacy, 700115 Iasi, Romania; maria.wolszleger@umfiasi.ro; 3 Department of Environmental and Food Chemistry, Faculty of Pharmacy, “Grigore T. Popa” University of Medicine and Pharmacy, 700115 Iasi, Romania; ionela.morariu@umfiasi.ro

**Keywords:** antimicrobials, wound infection, antimicrobial resistance, wound dressings

## Abstract

Wound healing is a complex, tightly regulated process essential for maintaining skin barrier function. Chronic wounds, often complicated by biofilm-forming bacteria and elevated oxidative stress, pose significant challenges in clinical management. The rise of antibiotic-resistant bacteria has further exacerbated the problem, limiting therapeutic options and complicating wound treatment. Traditional wound care approaches frequently fail to provide real-time accurate insights into wound status, leading to delayed or suboptimal treatments. Recent advancements in modern and smart wound dressings, which integrate various biosensors, different new drug delivery systems, and wireless communication technology, offers promising solutions for monitoring wound progression over time. These innovations enable early detection of adverse events such as bacterial infections and inflammation, facilitating more effective, on-demand treatment. This review highlights the current state of antibiotic-embedded wound dressings, discusses their limitations, and explores the potential of next-generation wound dressings incorporating microelectronic sensors for real-time monitoring and adaptive therapeutic responses to support healing and combat antimicrobial resistance.

## 1. Introduction

A skin wound is a pathological condition caused by disease, injury, or physiochemical damage. Based on the origin of the damage and the duration of healing, the wounds are classified as acute and chronic wounds [1]. The chronic wounds, such as diabetic ulcers, surgical wounds, and pressure injuries, are often trapped in the inflammatory stage so that they fail to progress toward healing and pose a greater mortality risk than commonly perceived [2,3].

For wound management, dressings are the primary strategy used in clinics; by definition, they are essential materials that cover and safeguard wounds, facilitating the healing process [4]. These dressings are crucial in shielding wounds from infections, absorbing exudate, and preserving a moist environment conducive to healing, as dry conditions can impede the process [5]. Dressings can be composed of various materials and serve multiple purposes, including medication delivery for infected wounds in some instances. Selecting the appropriate dressing can be complex, as it depends on the wound’s characteristics, patient requirements, and other variables [6]. While traditional dressings may suffice for dry wounds, advanced options like hydrogel and hydrocolloid dressings can maintain beneficial moisture levels for dry or necrotic wounds while preventing dehydration [7]. For infected wounds, it is vital to use dressings specifically designed for such cases, often incorporating antimicrobial properties to manage odor and inhibit bacterial spread, particularly when conventional antibiotics may be ineffective [8].

Wound management has been a crucial component of healthcare for many years, with infection prevention being essential for successful healing [9]. However, the rise of antibiotic resistance has emerged as a significant threat to the progress made in wound care over the past few decades [10]. This growing issue has caused concern throughout the medical field, leading to urgent requests for novel approaches to address what many professionals view as an impending healthcare crisis.

The problem of antibiotic resistance in wound care is particularly concerning due to the susceptibility of patients with open wounds to bacterial infections [11]. As bacteria become increasingly resistant to traditional antibiotics, medical professionals are finding their options for treating wound infections increasingly limited. This situation not only extends the time required for healing but also heightens the risk of serious complications that could potentially become life-threatening [12].

The term “critical colonization” has been used to describe the stage at which bacteria begin to adversely affect wound healing [13]. The wound microbiome consists of bacterial pathogens as well as fungi, which either interact with the bacteria and may increase antibiotic resistance, or which are even primary pathogens themselves [14]. Routine testing in pathology laboratories has primarily depended on culture methods to isolate potential pathogens from swabs, pus, or tissue biopsies. This process helps determine the likely identities of microorganisms and assess their antibiotic susceptibilities, providing guidance for informed antimicrobial treatment. Standardized approaches also facilitate global surveillance of antibiotic resistance [15]. Wounds often harbor polymicrobial communities, with *Staphylococcus aureus* being the most commonly isolated pathogen. Other frequently detected organisms include *Pseudomonas aeruginosa*, *Escherichia coli*, *Enterobacter cloacae*, *Klebsiella* species, *Streptococcus* species, *Enterococcus* species, and *Proteus* species [16]. Anaerobic bacteria, though often underestimated, are also prevalent, with common species such as *Peptostreptococcus, Prevotella, Porphyromonas, Bacteroides, Finegoldia magna*, and *Peptoniphilus asaccharolyticus* [17]. In addition, there are hundreds of microorganisms of different species attached and embedded in the extracellular matrix of biofilm [18].

The presence of multidrug-resistant bacteria in wounds significantly complicates the healing process, as persistent infections generate chronic inflammation and contribute to the formation of bacterial biofilms, which protect pathogens from the action of the immune system and antimicrobial treatment [10]. These persistent infections can lead to significant delays in tissue regeneration and chronic lesions. In addition to delayed healing, infections with resistant germs increase the risk of severe complications such as invasive infections (including sepsis), osteomyelitis, or even the need for amputation, particularly in patients with associated risk factors such as diabetes mellitus [19]. Clinically, antibiotic resistance severely limits available therapeutic options, often requiring the use of last-line antibiotics (like vancomycin or linezolid), which can be associated with higher toxicity and cost. Furthermore, empirical antibiotic therapy is becoming increasingly challenging as conventional regimens may no longer be effective against resistant strains, thereby delaying appropriate treatment and exacerbating patient outcomes [20].

In addition, multidrug-resistant infections often result in prolonged hospitalization, the need for repeated surgical interventions (e.g., debridements), and significantly increased treatment costs [21]. These patients may also become sources of infection for other patients or healthcare staff, requiring rigorous isolation and nosocomial infection control measures. The consequences of this trend go beyond individual patient care. The financial strain on healthcare systems, the possibility of widespread outbreaks of resistant infections, and the mental toll on patients dealing with persistent, non-healing wounds all contribute to the urgency of tackling this problem [22,23]. Apart from the economic impact, chronic wounds inflict a significant decrease in the patients’ quality of life.

Overall, the clinical burden of antibiotic resistance highlights the urgent need for a multifaceted approach to wound care, combining effective infection prevention strategies, the development of innovative antimicrobial technologies, and optimized therapeutic protocols to improve patient outcomes and mitigate the spread of resistant pathogens.

In response to this challenge, researchers, clinicians, and biomedical engineers are investigating a diverse array of novel strategies [24]. These encompass advanced wound dressings incorporating antimicrobial agents and innovative therapies that utilize the body’s immune system. The search for alternatives to conventional antibiotics has also prompted renewed interest in natural compounds [25], nanotechnology [26], and the utilization of beneficial bacteria to outcompete pathogenic microorganisms [27].

On the other hand, biofilm formation is a crucial step in the pathogenesis of chronic infections, including foreign body-related infections. It is important to note that difficult-to-heal wounds are chronic infections and that therapy should be directed toward local control of the biofilm (wound hygiene) [28], with systemic antibiotics reserved for cases where there is evidence of bacterial invasion of viable host tissue [29]. Wound biofilm formation is initiated by the attachment and proliferation of endogenous, exogenous, or ubiquitously present microorganisms on the wound surface [30]. These microorganisms aggregate into structured communities encapsulated within a protective polymeric matrix known as the extracellular polymeric substance (EPS), which enables them to adapt and survive in hostile environments. Biofilm development is driven by a complex interplay between genetic factors and environmental stimuli, such as surface characteristics and nutrient availability [31]. Under normal physiological conditions, the host immune system is capable of eliminating planktonic, free-floating bacteria [32]. However, in immunocompromised individuals or when bacterial growth is left unchecked, microbial proliferation leads to the establishment of highly organized biofilms shielded by the EPS matrix [33]. Polymicrobial biofilms are highly prevalent in chronic wounds and are associated with numerous defense mechanisms against environmental stress and host immune responses. Additionally, biofilms promote the secretion of inflammatory mediators that disrupt the normal wound healing cascade while perpetuating microbial persistence [34]. Within these biofilms, subpopulations of dormant, antibiotic-tolerant “persister” cells significantly contribute to treatment failures and recurrent infections [35]. Bacterial resistance mechanisms in this context include modifications to cell wall structure, biofilm-mediated protection, expression of efflux pumps, alteration or acquisition of antibiotic targets, and enzymatic degradation or modification of antimicrobial agents [15].

**Figure 1:**
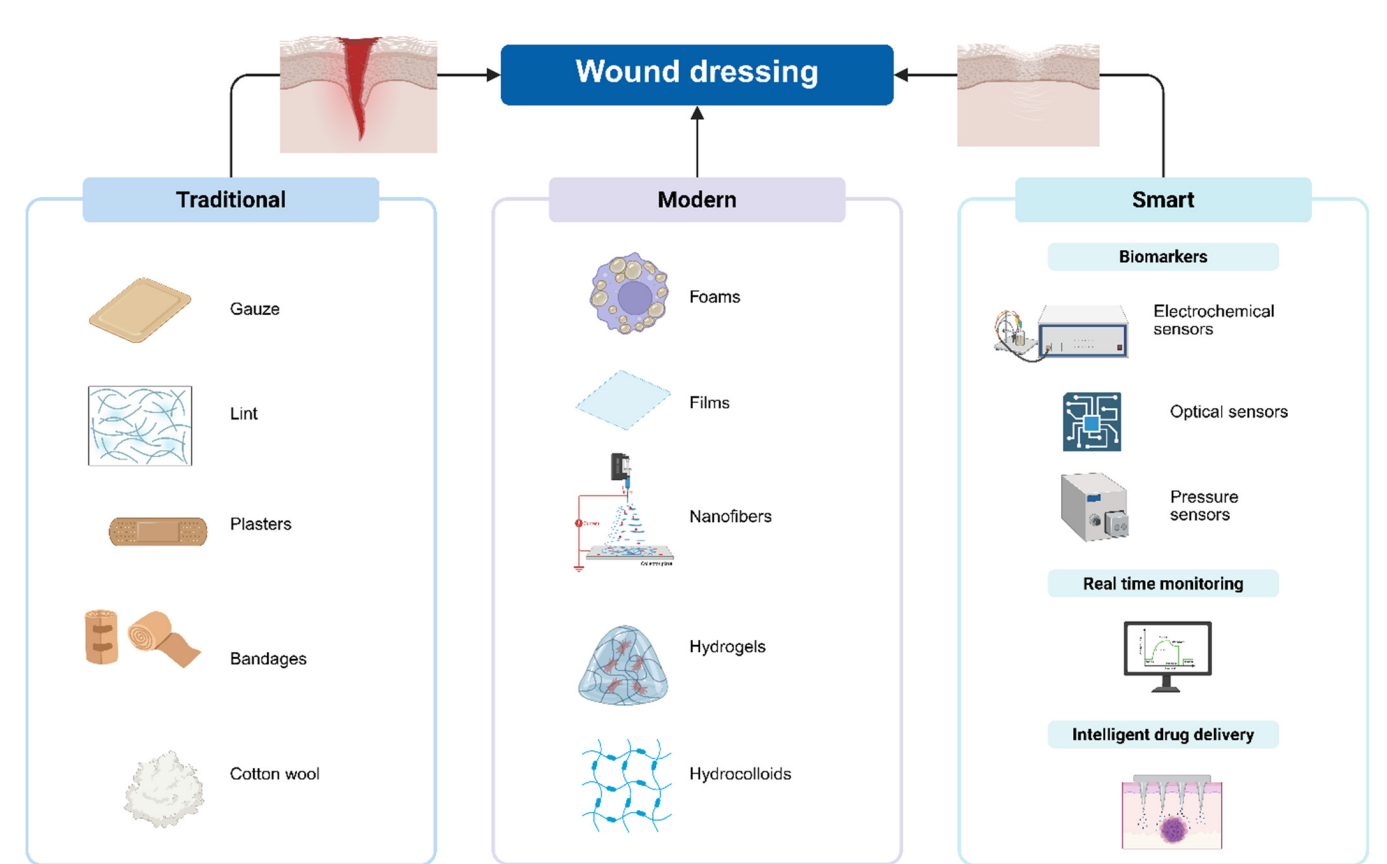
Schematic representation of wound dressing classification. Created in https://BioRender.com.

As the field of wound care reaches this critical juncture, it is evident that progress necessitates not only scientific innovation but also a paradigm shift in approaches to infection control. This work establishes the context for an examination of the innovative solutions being developed to address antibiotic resistance in wound care, presenting potential avenues for more efficacious and sustainable treatment modalities in the future. The aim of the present work is to explore the development and potential of antimicrobial smart dressings in improving wound care, while addressing the growing concern of antibiotic resistance. By integrating advanced materials and technology, these dressings provide localized and controlled release of antimicrobial agents, reducing the need for systemic antibiotics and minimizing the risk of infection (Figure 1). This paper examines how these innovative dressings can enhance wound healing, improve patient outcomes, and contribute to the fight.

## 2. Methodology

Comprehensive literature research was performed across PubMed, Scopus, and Web of Science databases using keywords related to wound dressings, nanoparticles, and natural compounds, specifically within the context of antimicrobial resistance. These terms and their respective synonyms were strategically combined using Boolean operators such as “AND” and “OR” to optimize search precision. The review included original research articles focused on the development of smart wound dressings. Only studies that investigated topical formulations, which were in vitro, ex vivo, or in vivo evaluated, were considered (Table 1).

## 3. Traditional Wound Dressings and Their Limitations in Combating Antibiotic-Resistant Bacteria

**Figure 2:**
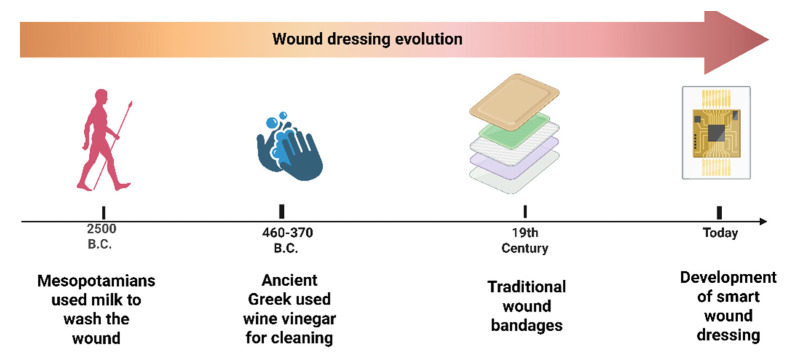
The evolution of wound dressings. Created in https://BioRender.com.

Traditional wound dressing products, including gauze, lint, plasters, bandages (natural or synthetic- Figure 1), and cotton wool, are dry and used as primary or secondary dressings for protecting the wound from contamination [59]. Ancient clay tablets reveal that wound bandaging dates back to 2500 BC (Figure 2). Archaeological evidence indicates that the earliest wound dressings were developed by the Egyptians. Their wound management system involved three steps: cleansing the wounds with milk, creating plasters, and applying bandages. They were the first people who applied honey to the wounds and invented the adhesive wound dressing [60]. In ancient Greece, between 460 and 370 BCE, Hippocrates employed wine or vinegar to cleanse wounds, followed by a treatment involving honey, oil, and wine. They also utilized wool that had been boiled in water or wine as a dressing. A significant advancement in antiseptic methods occurred during the 19th century, with the introduction of antibiotics to combat infections and reduce death rates [61].

Although traditional wound dressings have been used for decades to protect wounds, promote healing, and manage exudate, provide basic wound coverage and absorption, they have several limitations when it comes to combating antibiotic-resistant bacteria. These dressings often lack inherent antimicrobial properties; they primarily serve as physical barriers to external contaminants and facilitate moisture retention but lack any intrinsic antimicrobial activity, leaving wounds susceptible to bacterial colonization and infection [59]. Additionally, traditional dressings often suffer from poor drug permeability, necessitating frequent administration of antibiotics. This is particularly problematic in the presence of wound biofilms, which impede the entry of antibacterial agents. Also, the need for frequent changes can disrupt the healing process and increase the risk of introducing bacteria.

Maintaining optimal moisture balance is crucial for wound healing and preventing bacterial growth, yet many traditional dressings struggle in this aspect. While acting as a physical barrier, these dressings provide only passive protection without actively combating bacteria or promoting healing. Some may even adhere to the wound bed, causing pain and potential tissue damage upon removal [62]. The adherence of these dressings can lead to significant pain during dressing changes, which can be distressing for patients. While the hydrophilic dressings often fail to effectively manage wound exudate, creating conditions favorable for bacterial growth, in opposition, some newer dressings with hydrophobic properties can cause excessive dryness, disrupting the moisture balance necessary for healing [63].

Antibiotic-resistant bacteria often form biofilms, which traditional dressings cannot effectively penetrate or disrupt [64]. They do not address the dynamic and evolving biological microenvironment of infected wounds, particularly the formation of resilient bacterial biofilms, which are key contributors to chronicity and therapeutic failure [32]. In the presence of multidrug-resistant organisms, the reliance on systemic antibiotics in conjunction with traditional dressings often proves inadequate, as resistant strains evade eradication through biofilm-mediated protection and altered metabolic states [65].

Oxygen permeability is another concern, as some dressings may not allow sufficient oxygen to reach the wound, potentially slowing healing and creating an environment conducive to anaerobic bacterial growth [66].

Unlike modern advanced dressings, traditional options cannot provide controlled release of antimicrobial agents over time [67]. This can lead to premature drug release or unintended drug activation, reducing treatment efficacy and potentially contributing to resistance. Even when impregnated with antibiotics, they may be ineffective against resistant strains. Also, the limited customization options of traditional dressings, which often come in standard sizes and shapes, make it challenging to address wounds with complex geometries or locations [62].

Additionally, classical dressings also cannot be used to modulate the inflammatory milieu or provide a local and sustained antimicrobial effect, which are both critical for treating chronic infections without causing antibiotic resistance [68]. The fact that they fail to address microbial virulence factors, quorum-sensing mechanisms, or hypoxic wound status makes the task of antimicrobial approaches even more difficult. Thus, the prolonged application of such traditional dressings in antibiotic-resistant infected wounds not only promotes wound-healing retardation but may also cause improper selecting and transmission of drug-resistant phenotypes [69,70]. These drawbacks stress the compelling necessity for the implementation and integration in clinics of next-generation dressings featuring multifunctionality such as active antimicrobial effect, biofilm disruption, and enhanced tissue regeneration.

The limitations of traditional dressings have catalyzed the development of innovative materials and technologies aimed at addressing the complex microenvironments characteristic of chronic and infected wounds [71]. One promising strategy involves the integration of stimuli-responsive systems, wherein dressings release antimicrobial agents selectively in response to local triggers such as pH shifts, enzymatic activity, or temperature changes associated with infection [72]. These “smart” materials enable on-demand drug delivery while minimizing the risk of antimicrobial resistance by avoiding continuous subtherapeutic exposure [73].

In order to counter these shortcomings and limitations, nanotechnology continues to offer transformative opportunities in wound care. Nanoparticle-based systems, including silver, copper oxide, zinc oxide, and carbon-based nanostructures, exhibit potent antimicrobial activities through multiple mechanisms, such as the generation of reactive oxygen species (ROS), disruption of microbial membranes, and interference with bacterial metabolic pathways [74,75]. Recent advances in biodegradable nanocomposites have further enabled the incorporation of antimicrobial nanoparticles into biocompatible polymer matrices, thereby reducing cytotoxicity concerns while maintaining efficacy [76].

In addition, the incorporation of natural antimicrobial agents into wound dressings has emerged as an attractive strategy to overcome the limitations associated with traditional antibiotics and synthetic antimicrobials. Plant-derived compounds such as essential oils (e.g., lavender oil), flavonoids (e.g., quercetin, curcumin), alkaloids, and polyphenols exhibit broad-spectrum antimicrobial activities by targeting bacterial cell membranes, disrupting metabolic pathways, and inhibiting biofilm formation, all without promoting significant resistance [74,77]. Furthermore, natural polymers such as chitosan and honey-derived substances offer intrinsic antibacterial properties alongside immunomodulatory and antioxidant benefits, making them ideal candidates for multifunctional wound care systems [78].

## 4. The Concepts of Modern Dressings

Since traditional dressings fail to provide a moist environment to the wound, they have been replaced by modern dressings with more advanced formulations: films, foams, nanofibers, hydrogels, and hydrocolloids (Table 2).

### 4.1. Films

Film dressings are highly versatile and effective for treating a variety of superficial wounds, such as skin grafts, surgical wounds, and minor burns. Films are thin, transparent, adhesive materials designed to maintain a moist wound environment, reduce the risk of infection, and facilitate easy wound monitoring [79]. These dressings create an optimal moist environment that promotes healing and serves as a protective barrier against bacterial contamination.

A film dressing is a transparent, thin polymer layer that acts as a shield against external contaminants and physical damage while ensuring a moist healing environment for the wound. They can be customized to have properties such as adhesion, gas permeability, and antimicrobial capabilities. However, one of the challenges of using film dressings is that their removal can be difficult and may result in damage to newly formed tissue [100]. Additionally, since films do not absorb or remove exudate from the wound, fluid accumulation can potentially harm newly differentiated keratinocytes. To address these concerns, it is important to consider dressings that can be easily removed without causing tissue damage, especially for chronic wounds [101].

### 4.2. Hydrocolloids

Introduced in 1982, hydrocolloid dressings are composed of a flexible wafer with an inner layer of hydrophilic gel, made from a combination of gelatin, pectin, sodium carboxymethylcellulose, and polyisobutylene, backed by a film [102]. Hydrocolloid dressings are most commonly associated with the treatment of chronic wounds such as leg ulcers and pressure ulcers. They can also be used with good effect for the treatment of a variety of acute wounds, where their ability to facilitate debridement, absorb excess fluid, and provide a barrier to infection is equally valuable [103].

These occlusive coverings have been demonstrated to create a moist, low-oxygen environment that encourages autolytic debridement. While allowing gas exchange and being partially permeable to vapor, they may cause skin softening if applied to wounds with heavy discharge. However, these dressings are commonly used in wound care for their ability to form a gel-like protective layer upon contact with wound exudate, creating a moist environment that promotes healing [104]. This moist environment is beneficial for wound healing but can also be conducive to bacterial growth, which necessitates the incorporation of antimicrobial agents to prevent infections. For instance, a study demonstrated that the incorporation of CM11 antibacterial peptide into hydrocolloid dressings showed significant antibacterial activity against multidrug-resistant (MDR) strains of *Pseudomonas aeruginosa, Staphylococcus aureus*, and *Escherichia coli* [89].

Various specific types of hydrocolloid dressings have been developed, differing in dimensions, form, fluid absorption capacity, and intended application [90,91].

### 4.3. Foams

Foam dressings are widely used in wound care due to their ability to absorb exudate and maintain a moist environment conducive to healing. Their non-adhesive quality potentially reduces damage to the wound and surrounding skin during removal. This may prevent further tissue harm and allow undisturbed healing, possibly decreasing pain and discomfort [5]. It is also believed that the moist environment fostered by foam dressings promotes wound healing. For example, this study has demonstrated that antimicrobial foams containing natural extracts like *Centella asiatica* can significantly improve wound recovery rates [82].

Additionally, foam provides a flexible protective barrier against contamination while allowing movement. Typically, foam dressings are secured with gauze and tape. Some are waterproof with adhesive borders, eliminating the need for additional covering. Most foam dressings consist of highly absorbent polyurethane. For example, lignin-based polyurethane foams with silver nanoparticles are biodegradable and exhibit excellent antimicrobial properties without significant cytotoxicity [83].

Antimicrobial foam dressings incorporate agents like polyhexamethylene biguanide (PHMB), silver nanoparticles, and natural extracts to inhibit microbial growth [84]. For instance, PHMB-treated foams have shown significant efficacy in reducing methicillin-resistant *Staphylococcus aureus* (MRSA) [105]. Similarly, silver nanoparticle-infused foams exhibit over 95% bactericidal efficacy against *Escherichia coli* and *Staphylococcus aureus* [85]. Furthermore, this study underscores the synergistic potential of combining mangosteen extract, which possesses antibacterial properties, with curcumin extract, known for its anti-inflammatory and antioxidant activities. The integration of these natural extracts contributes to enhanced antibacterial and anti-inflammatory efficacy [106].

Thus, specialized antimicrobial foams like those containing methylene blue and gentian violet are effective in managing wounds in pediatric patients, providing antibacterial coverage and promoting healthy wound edges [86].

### 4.4. Hydrogels

In 1960, Wichterle and Lim successfully prepared a hydrogel by polymerizing methyl-2-hydroxyethyl methacrylate and made the first contact lens in history, thus starting the application research on hydrogels. Hydrogels are polymer materials with a three-dimensional network structure, formed by polymer chains linked through physical or covalent bonds [94]. Due to their exceptional properties, hydrogels have gained significant attention, particularly in biomedical fields. Their high concentration of hydrophilic groups allows them to absorb water up to hundreds of times their own mass, resulting in excellent hydrophilicity, water swelling, and retention properties. The high water content of hydrogels enables them to cool wounds and alleviate the warmth associated with inflamed tissue [107]. Their biocompatibility and cell adhesion properties allow direct contact with injuries, reducing body fluid loss and preventing secondary infections, thus aiding wound healing. Hydrogels can be applied to various medical devices for drug release, including catheters, central venous catheters, joint implants, and dental implants. They can also be used in textile applications and wastewater treatment by grafting hydrogel monomers onto fibers, coating fabrics, or creating intelligent textiles and dressings [108]. However, the widespread use of antibiotic-loaded traditional hydrogels may contribute to the development of antibiotic resistance, diminishing their effectiveness. To address this issue, researchers developed antibacterial hydrogels, which combine the benefits of hydrogels with antibacterial properties. These advanced materials feature simple preparation processes and diverse structures, leading to the development of various innovative antibacterial hydrogels [95,109]. As an illustrative example of an advanced antimicrobial hydrogel dressing, a multifunctional formulation has been developed comprising Cypate-conjugated antimicrobial peptides (AMP-Cypates), liposome-encapsulated perfluorodecalin, and recombinant type III collagen. AMP-Cypates demonstrate potent antibacterial activity, leveraging the combined effects of antimicrobial peptides (AMPs), photothermal therapy (PTT), and photodynamic therapy (PDT). The inclusion of perfluorodecalin-loaded liposomes serves as an oxygen delivery system, effectively alleviating hypoxic conditions at the wound site and enhancing the therapeutic efficacy of PDT. Concurrently, recombinant type III collagen facilitates tissue regeneration and accelerates wound healing. Collectively, this hydrogel dressing exemplifies a versatile platform that integrates multiple antimicrobial strategies to efficiently eliminate bacterial infections and promote the healing of chronic wounds [110].

Additionally, Jiang et al. [111] incorporated clindamycin into glycerol-based hydrogels and fabricated fiber-based dressings. The resulting hydrogel fabric dressing demonstrated remarkable efficacy in promoting the healing of infected wounds, achieving complete skin closure in rats within 14 days. Such rapid healing is not typically attainable with conventional hydrogel dressings, thereby presenting a promising new approach for the advancement of wound care therapies.

### 4.5. Nanofibres Dressings

Nanofiber dressings constitute a notable innovation in wound management, providing a range of benefits including accelerated healing, effective infection control, and minimized patient discomfort. Their structural similarity to the extracellular matrix (ECM) [96], coupled with the incorporation of bioactive agents, renders them a highly versatile and efficacious solution for treating diverse wound types, such as diabetic ulcers, burns, and chronic wounds [112]. Among natural polymers: chitosan [97], alginates [113], collagen [114], gelatin [115], and silk [116] are the most frequently utilized in the fabrication of nanofibrous scaffolds. In contrast, commonly employed synthetic polymers include polylactic acid (PLA) [117], poly(lactic-*co*-glycolic acid) (PLGA) [118], polycaprolactone (PCL) [119], and poly(caprolactone-lactide) copolymers.

Alginate is a naturally occurring, anionic biopolymer extracted from various brown algae species, including *Phaeophyceae*, *Ascophyllum nodosum*, *Laminaria hyperborea*, *Laminaria japonica*, *Macrocystis pyrifera*, and *Laminaria digitata* [120]. It is widely available, biocompatible, and non-toxic. Wound dressings made from alginate help maintain a moist environment and reduce the risk of bacterial infections, both of which are critical for effective wound healing. Alginate gels form in the presence of trivalent ions like Al and Fe. The binding of these trivalent cations with alginate results in a more compact gel network compared to the interaction with divalent cations [121]. Alginate dressings are created through the ionic cross-linking of their solution with ions such as calcium, magnesium, barium, lead, cadmium, cobalt, zinc, nickel, manganese, or strontium, to form a gel [122]. These dressings are capable of absorbing wound exudate in their dry form, transforming into gels that provide a physiologically moist environment for dry wounds, reducing bacterial infection, and promoting faster re-epithelialization and granulation tissue formation [123].

Although nanofiber dressings represent a significant advancement in wound care due to their high surface area, tunable porosity, and ability to mimic the extracellular matrix (ECM), several limitations restrict their effectiveness, particularly against antibiotic-resistant infections. Also, concerns regarding biocompatibility and cytotoxicity remain significant barriers to clinical translation. Although they ensure enhanced cellular adhesion and controlled drug release, conventional nanofibrous scaffolds have poor inherent antimicrobial activity unless functionalized with bioactive agents [124]. Moreover, sustained release of antibiotics or antimicrobial drugs from nanofibers tends to create subtherapeutic exposure for long durations, indirectly promoting the selection of resistant bacterial populations and the development of tolerance mechanisms [125]. Another critical challenge is the susceptibility of nanofiber matrices to biofilm colonization. As the bacteria attach, the dense network of nanofibrous scaffolds’ architecture can create a microenvironment that protects biofilms from host immunity responses and antimicrobial therapy [126].

Furthermore, the manufacturing processes of fibers, such as electrospinning, typically involve the employment of organic solvents such as hexafluoroisopropanol (HFIP) or dimethylformamide (DMF), which may leave behind residual solvent traces in the final product if not properly eliminated and have the ability to induce cytotoxic reactions upon use [127]. Furthermore, the incorporation of antimicrobial agents, such as metal nanoparticles (e.g., silver, zinc oxide) or synthetic antibiotics, within nanofibrous scaffolds can disrupt cellular homeostasis, trigger oxidative stress, and suppress fibroblast cell proliferation and migration, which are all critical wound healing functions [128]. Surface chemistry and degradation products of the fibers also influence cellular response. For instance, certain synthetic polymers like polycaprolactone (PCL) and polylactic acid (PLA), although well recognized as biocompatible, produce acidic degradation products that can have a negative effect on the wound microenvironment, particularly in chronic wounds with impaired buffering capacity [129]. Additionally, very dense nanofiber mats can hinder oxygen and nutrient supply to the underlying tissue, resulting in additional hypoxia and compromising healing [130]. Thus, although nanofiber dressings offer promising features, their clinical efficacy against antibiotic-resistant infections, biocompatibility, and cytotoxicity remain significant barriers to clinical translation.

## 5. Transition from Modern Dressings to Smart Dressings

Antimicrobial smart dressings are emerging as a crucial innovation in wound care, particularly in the fight against antibiotic resistance. These dressings incorporate antimicrobial agents directly into the dressing material, providing localized treatment and reducing the need for systemic antibiotics, which are often associated with the development of resistant bacterial strains [22].

An ideal wound dressing must provide two crucial functions: preserving wound moisture and safeguarding against bacterial infection [131]. One of the primary causes of delayed wound healing is the formation of bacterial biofilms, which occur when bacterial cells accumulate, embed, and proliferate at the wound site. Actual dressings with antibacterial properties offer temporary protection against biofilm formation by passively releasing antibacterial agents [132].

Concurrently, extensive research is underway to develop a new generation of wound dressings capable of real-time wound condition monitoring and on-demand therapeutic delivery [133]. The creation of a dressing that can detect bacterial film colonization would be advantageous for healthcare professionals.

Recent studies have focused on developing intelligent wound dressings that respond to physical (pH, light, and temperature) and biochemical (enzymes and bacterial toxins) indicators of wound infections [134]. These advanced dressings can monitor and assess wound conditions in real-time while releasing encapsulated antibacterial drugs in a controlled manner [135].

### 5.1. Wound-Related Biomarkers

Early diagnosis of wound infections that aid in the assessment of the stage of wound infection is gaining interest for development of a new biomarker. Smart dressings equipped with integrated sensors represent a promising technology that could revolutionize wound care by enabling continuous, real-time monitoring of the healing process [136]. By incorporating miniaturized biosensors directly into the dressing, valuable information can be obtained regarding key parameters such as pH, temperature, humidity, oxygen saturation, enzymes (e.g., lysozyme, α-amylase, protease), metabolites (uric acid, lactic acid, volatile organic compounds, nitrogen peroxide and nitric oxide), and cytokines, without the need to remove the dressing [137]. These sensors are designed to detect low concentrations of bacterial cells and other infection markers, ensuring high sensitivity and specificity. For example, sensors can detect bacteria concentrations as low as 10^2^ CFU/mL and provide stable readings over extended periods [36].

#### 5.1.1. Electrochemical Sensors

A promising area of development is the integration of electrochemical sensors to detect and quantify bacterial load in the wound bed. These sensors can be designed to identify specific bacterial species or to provide a general measure of microbial proliferation. By continuously monitoring bacterial presence, smart dressings could enable early detection of infection and facilitate more timely, targeted antibiotic interventions [138]. Advanced electrochemical sensors can simultaneously measure multiple analytes such as pH, uric acid, myeloperoxidase (MPO), and CRP, offering a comprehensive assessment of the wound environment [139].

MPO activity in a real sample matrix, wound fluid, is considered one of the potential sources of several wound biomarkers that are easy for specimen collection and provide several informative biomarkers for wound monitoring. Some reports of electrochemical biosensors for MPO have been reported in the literature [140]. A portable sensor has been developed to detect MPO activity in wound exudate samples. This sensor uses glucose oxidase to produce hydrogen peroxide, which is then catalyzed by a Prussian blue/MXene composite. The data are wirelessly transferred to a smartphone, making it suitable for resource-limited settings. Another group of researchers created an MPO-based in vitro test strip sensor [52]. In this case, the results showed a good correlation between sensor current and MPO concentration, which proved the ability of the novel in vitro test strip sensor to reliably detect wound infection at an early stage.

Current developments include electrochemical sensors for pyocyanin detection that offer promising solutions for identifying *Pseudomonas aeruginosa* infections. One such sensor utilizes a multiwalled carbon nanotube nanocomposite, demonstrating high selectivity and sensitivity across various sample matrices [53]. Additionally, a disposable electrochemical sensor detects pyocyanin in wound fluid, delivering rapid results without the need for sample preparation, making it an effective option for point-of-care diagnostics. These advancements enhance the potential for efficient and timely detection of infections in clinical settings [54].

#### 5.1.2. Optical Sensors

Another innovative approach involves incorporating optical sensors into smart dressings to assess tissue oxygenation and perfusion [141]. Techniques such as near-infrared spectroscopy [142] or fluorescence-based methods [143] can be used to measure oxygen levels in the wound, providing crucial insights into the healing environment. Adequate tissue oxygenation is essential for proper wound healing, and real-time monitoring of this parameter could help healthcare providers optimize treatment strategies and identify potential complications early. The development of portable optical biosensors has revolutionized point-of-care diagnostics by enabling rapid and accurate detection of infections at the patient’s bedside. These devices are designed to be user-friendly, cost-effective, and capable of providing immediate results, which is essential for effective wound management [144].

Colorimetric sensors change color in response to specific biochemical markers. For instance, a 3D-printed colorimetric indicator contains a pH-sensitive dye that detects carbon dioxide production from wound pathogens by changing color from blue to yellow. The sensor accurately reported the minimum inhibitory concentration of antibiotic against *Pseudomonas aeruginosa* in a sealed 96-well plate. This sensor holds promise for monitoring wound infection treatment efficacy [37].

Fluorimetric detection of wound status is typically conducted by measuring the fluorescence intensity of a sample using fluorescence spectroscopy. This method is regarded as one of the most important techniques due to its simplicity and good sensitivity. Mirani et al. [56] developed a multifunctional alginate hydrogel-based dressing that incorporates an array of porous, color-changing pH sensors for detecting bacterial infections. Thanks to advanced 3D printing technology and by using a broad pH-sensing range (which can be achieved by simply adjusting printing parameters), the fiber-based sensor array can be easily obtained. Moreover, the sensor’s response rate to pH can be tailored by altering the fiber diameter during the printing process. The evaluations showed that this smart dressing accurately detects pH changes in pig skin colonized with varying densities of *P. aeruginosa*, effectively indicating bacterial infection and aiding in treatment decisions [56].

#### 5.1.3. Pressure Sensors

Smart dressings can also integrate pressure sensors to monitor the pressure applied to the wound and detect early signs of pressure ulcers. This is particularly beneficial for bedridden or mobility-impaired patients [145]. These sensors provide quantitative data on applied pressure, pressure gradients, and fluctuations, aiding clinicians in correlating medical outcomes with specific treatment regimes. By alerting caregivers to areas of sustained pressure, these dressings can facilitate timely interventions, such as repositioning or the use of pressure-relieving devices, potentially preventing the development of severe pressure injuries [146].

Prototypes of electronic layers with force-sensitive pressure sensors and integrated temperature and humidity sensors have been designed to monitor wound site activities. These layers can detect external pressure and provide real-time monitoring of temperature and humidity, essential for managing pressure ulcers. Furthermore, the device is also capable of sending an alert to the client station display whenever the temperature, humidity, and pressure values go beyond their set threshold [57].

Data collected by these integrated sensors can be wirelessly transmitted to smartphone applications or to healthcare providers’ monitoring systems for continuous analysis. Advanced algorithms and machine learning techniques can be applied to interpret sensor data, providing clinicians with valuable insights and personalized treatment recommendations. This seamless integration of sensor technology with data analysis has the potential to significantly improve wound care outcomes and reduce the medical costs associated with chronic wound treatment [58].

As sensor technology continues to advance, future smart dressings may incorporate even more sophisticated diagnostic capabilities. For example, the integration of DNA-based sensors could enable rapid identification of genetic markers associated with antibiotic resistance, guiding more precise antimicrobial therapy. These sensors use oligonucleotide probes modified with redox labels (e.g., methylene blue) attached to electrodes. The binding of target DNA changes the probe’s structure, affecting electron transfer and generating a measurable signal [147]. Wang et al. [55] in their study, the E-DNA (ultrasensitive electrochemical DNA) sensors based on isothermal strand-displacement polymerization reaction (ISDPR) have been successfully used to detect specific antibiotic resistance genes, such as the mecA gene in methicillin-resistant *Staphylococcus aureus* (MRSA). The proposed DNA biosensor could offer excellent analytical performance for the detection of mecA gene and provide a new electrochemical method for the early diagnosis of drug-resistant bacteria.

Additionally, the development of flexible and stretchable electronics may lead to smart dressings that can adapt to the changing contours of the wound while maintaining precise detection capabilities throughout the entire healing process.

### 5.2. Wound Dressing with Intelligent Drug Delivery

Effective wound healing, particularly for chronic wounds, relies on the timely and controlled delivery of bioactive factors and drugs. Research has demonstrated their significant impact on skin regeneration. Intelligent wound dressings, which integrate real-time wound monitoring with dynamic drug release, have emerged as a promising solution [148]. These systems are attracting substantial research interest and demonstrating significant progress in actively treating wounds and accelerating healing [72,149].

Traditional dressings typically release antimicrobials through passive diffusion, which can result in overuse and the development of resistance. In contrast, newer dressing designs, such as this study [38] presents a straightforward method for synthesizing a high-strength, non-releasing antimicrobial hydrogel dressing composed of poly(ionic liquid)/polyvinyl alcohol (PVA), achieved through a combination of chemical polymerization and physical cross-linking. The resulting hydrogel demonstrated broad-spectrum antimicrobial efficacy against a range of microorganisms, including *Escherichia coli*, *Staphylococcus aureus*, *Bacillus subtilis*, *Candida albicans*, *Aspergillus niger*, *Aspergillus oryzae*, and *Rhizopus* species. In a murine wound model, the hydrogel dressing significantly accelerated cutaneous wound healing. Histological analysis after 15 days revealed enhanced epidermal regeneration compared to the control group, indicating its strong potential for promoting effective skin repair.

Additionally, some dressings utilize a physical sequestration approach, binding bacteria to the dressing and removing them during dressing changes. This method helps prevent prolonged inflammation and reduces the potential for resistance [150,151].

Some smart dressings release antimicrobials only in the presence of pathogenic bacteria, reducing unnecessary antibiotic use and minimizing resistance development. For example, dressings with UV-photocrosslinkable methacrylated gelatin (GelMA) release antimicrobials in response to toxins from methicillin-resistant *S. aureus* and *P. aeruginosa* [39]. In another study, dressings doped with calcium phosphate nanoparticles (CaP NPs) can release antimicrobial agents in response to the alkaline pH of infected wounds, ensuring targeted treatment [40]. For severely infected wounds, Huang et al. [41] synthesized self-healing hydrogels (QCS/OD/TOB/PPY@PDA) with electrical conductivity and antioxidant properties. These hydrogels utilize a pH-responsive release system, where tobramycin (TOB) is released on-demand due to bacterial growth acidity, enabled by Schiff base cross-links between TOB and oxidized dextran (OD). This smart release mechanism avoids antibiotic overuse. The hydrogels, composed of quaternized chitosan (QCS), oxidized dextran, TOB, and PPY@PDA NWs, demonstrated potent antibacterial activity against multiple bacteria, including drug-resistant strains, lasting up to 11 days. In vivo studies showed improved wound healing, reduced inflammation, and enhanced tissue regeneration in infected burn wounds.

Smart hydrogels are engineered to release drugs in response to specific environmental stimuli, including pH changes or enzyme presence. Yang et al. [42] created an innovative wound dressing that allows for in situ bacterial detection and subsequent photodynamic antibacterial therapy. This injectable hydrogel, based on carboxymethyl chitosan and oxidized sodium alginate, utilizes MUG (4-methylumphulone beta-D-glucoside) and up-converted nanoparticles coated with titanium dioxide UCNPs@TiO_2_. Bacterial detection is achieved through the blue fluorescence emitted when MUG reacts with pathogen-associated enzymes. In vivo studies in rats demonstrated the dressing’s ability to effectively combat bacterial infections and promote wound healing. Furthermore, a smartphone-linked detection system was developed, enabling rapid pathogen identification with a sensitivity of 10^3^ CFU, suggesting the dressing’s suitability for clinical wound infection diagnosis and treatment. Zhang et al. [152] developed a pH-responsive hydrogel dressing (BPPRH) by grafting acrylic acid (AA) onto the molecular chains of a bacterial cellulose (BC) network through copolymerization. These “smart” hydrogels were subsequently loaded with curcumin (Cur) to endow them with antimicrobial functionality. The structural characterization revealed a three-dimensional porous architecture capable of modulating drug release in response to variations in pH typical of wound environments. Drug release kinetics under different physiological conditions were consistent with logistic and Weibull models, indicating a controlled and stimulus-responsive mechanism. Antimicrobial testing demonstrated that the BPPRH-Cur dressings exhibited substantial inhibitory activity against *Escherichia coli, Staphylococcus aureus,* and *Pseudomonas aeruginosa*, primarily through mechanisms involving membrane disruption and bacterial cell lysis.

Shi et al. [43] developed nanoparticle-based wound dressings that release antibiotics and anti-inflammatory drugs in response to light or temperature. Using electrospinning, they created an amphiphilic nanofiber membrane (PLGAV-CuS/PVAM) from polyvinyl alcohol (PVA), poly(lactic-*co*-glycolic acid) (PLGA), and copper sulfide nanoparticles (CuS NPs), incorporating mupirocin (M) and valsartan (V). This membrane enabled controllable gradient drug release, demonstrating low toxicity and good biocompatibility. In vitro, mupirocin was released slowly in a hydrophilic environment for antibacterial action. The membrane’s photothermal effect regulated valsartan release, reducing inflammation and enhancing antibacterial efficiency (96.3% against *E. coli* and 97.8% against *S. aureus*). These patches, with their sustained-release, antibacterial, and anti-inflammatory properties, highlight the potential of nanofibers in biomedicine.

In a recent study, Deng et al. [153] introduced a wireless, flexible smart wound dressing capable of monitoring matrix metalloproteinase-9 (MMP-9), a key biomarker in chronic wound environments. The system integrates a highly sensitive radio frequency sensor, enabled by a bioresponsive hydrogel functionalized with bioactive peptide sequences. Leveraging a flexible inductive-capacitive (LC) circuit design, the dressing facilitates real-time, in situ wound assessment. In addition, the hydrogel enables the controlled release of silver nanoparticles (AgNPs), which demonstrate potent antimicrobial activity against common wound pathogens such as *Escherichia coli* and *Staphylococcus aureus*. The device also achieved accurate MMP-9 detection in exudates from diabetic foot ulcer (DFU) patients, with performance comparable to standard fluorescence-based assays.

Wound management is evolving from traditional hospital care to the use of multifunctional, closed-loop smart dressings. These dressings provide long-term real-time monitoring, fast diagnosis, and personalized treatments, leading to improved wound management and positive outcomes for both patients and clinicians. Continuous data from these sensors allow for personalized wound care strategies, optimizing treatment plans based on the specific needs of the patient [154].

## 6. Types of Antimicrobial Agents Used in Smart Dressings

The risk of infection becomes critical after an injury resulting in a wound, as the wound environment provides an ideal setting for bacterial growth. Preventing bacterial invasion is essential for efficient wound healing. Conventional systemic treatments to combat infection often require high concentrations of antibiotics, which can lead to cytotoxic side effects. As a result, one of the primary strategies for effective wound treatment is the controlled delivery of antibiotics directly to the wound area through wound dressings. These dressings can prevent wound infections without the systemic toxicity associated with oral or intravenous antibiotics, thus reducing morbidity, mortality, and healthcare costs [155].

**Figure 3:**
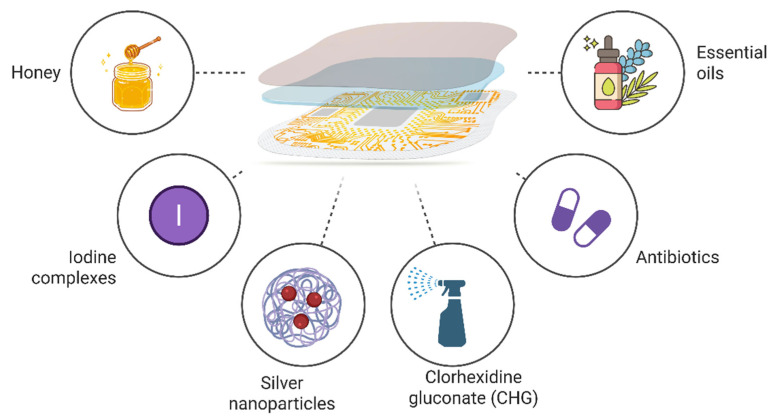
Antimicrobial agents used in smart dressings. Created in https://BioRender.com.

However, further research is required to optimize the implementation of nanoparticle-based therapies and their clinical translation. We can advance our understanding of multidrug resistance by addressing these research gaps. The development of evidence-based strategies to improve patient outcomes for wound infections. Finally, the global burden of antimicrobial resistance in healthcare settings can be mitigated [156] (Figure 3).

### 6.1. Antibiotics

Wound dressings that include antibiotics should offer effective antibacterial performance, maintain an appropriate moist environment, and be non-toxic to host tissues. Common antibiotics used in wound healing include aminoglycosides (gentamicin, streptomycin), beta-lactams (ampicillin, ceftazidime, cefazolin), glycopeptides (vancomycin), quinolones (ciprofloxacin, levofloxacin), sulfonamides (sulfadiazine), and tetracyclines (doxycycline, tetracycline hydrochloride) [157,158]. These antibiotics work in various ways, such as inhibiting bacterial cell wall synthesis (beta-lactams and glycopeptides), interfering with the synthesis of key metabolites (sulfonamides), blocking protein synthesis pathways (aminoglycosides and tetracyclines), and inhibiting nucleic acid synthesis (quinolones).

The effectiveness of an antibacterial wound dressing that delivers antibiotics depends on factors such as the drug release profile, the physicochemical properties of both the drug and the polymeric material [159]. For optimal wound infection treatment, an initial burst release (about 60% of the drug within the first 5 h) is required, followed by a controlled release to maintain the drug’s therapeutic effect [148]. In the literature, commonly used antibiotics for antibacterial wound dressings include gentamicin, ciprofloxacin, tetracycline, and ampicillin. Hu et al. [44] developed sustained antimicrobial wound dressings by covalently bonding ciprofloxacin HCl (CIP) and gentamicin sulfate (GS) to a carboxymethyl chitosan (CMC) and collagen (COL) polymer matrix. The resulting dressings, CMC-COL-CIP and CMC-COL-GS, achieved controlled release of antibiotics via amide bond formation. In vitro and in vivo studies confirmed effective and prolonged antimicrobial activity.

As antibiotic resistance continues to rise, with infection-causing strains gradually developing tolerance, there has been increasing interest in exploring a wide range of bioresources. These alternatives primarily include herbs, but also extend to animal products and nanoparticles containing mineral ingredients [160]. Numerous natural agents with antimicrobial activity against polymicrobial wound infections have been documented in the literature. These agents demonstrate bactericidal effects, particularly at the biofilm level, targeting both the initial and advanced stages of wound infection [161].

### 6.2. Honey

The use of honey as an antimicrobial agent is well-established in modern wound care, with medical-grade honey incorporated into various commercially available dressings [162]. These dressings provide antimicrobial and anti-inflammatory benefits by promoting autolytic debridement, maintaining a moist wound environment, inhibiting bacterial growth, promoting healing, and deodorizing the wound. However, research findings on their overall effectiveness have been mixed [163].

Honey is both bactericidal and antifungal, effective against a wide range of bacterial strains, including both Gram-positive and Gram-negative bacteria, as well as some yeasts. It is particularly useful for controlling bacterial strains resistant to conventional antibiotics. The antimicrobial action of honey is both mechanical and enzymatic. Similar to sugar pastes, honey inhibits bacterial growth due to its high osmolarity, where the concentration of sugars draws water from the wound environment [164]. This osmotic effect also helps maintain a moist wound environment by stimulating fluid transfer from surrounding tissues. Although this dilutes the honey, its antibacterial properties remain intact.

Honey is applied topically in various forms, such as ointments, for packing cavities, or impregnated in hydrogel or alginate dressings [165]. When used as an ointment, honey quickly dilutes due to the absorption of wound exudate and its increased fluidity at body temperature, which may require more frequent dressing changes to ensure continued effectiveness [166].

### 6.3. Essential Oils

Essential oils (EOs), which are extracted from plants through distillation and other methods, are widely used in the food, medicine, and cosmetics industries due to their antibacterial, antioxidant, anti-inflammatory, anti-allergic, antiviral, and regenerative properties [167]. Several studies have shown that the antimicrobial action of EOs, when incorporated into wound dressings, can be attributed to their various active constituents, such as cinnamaldehyde, geraniol, thymol, menthol, and carvacrol [168]. The composition and concentration of these compounds depend largely on the extraction method (e.g., hydrodistillation, microwave-assisted extraction, steam distillation, microwave-generated hydrodistillation, microwave steam diffusion, and ultrasound-assisted extraction) and the source of the sample [169].

EOs primarily contain terpene groups, which contribute to their antibacterial properties. The antibacterial mechanism of action is thought to involve an increase in membrane permeability or cytoplasmic leakage due to interactions with phospholipids in the cell wall [170]. Damage to the cytoplasmic membrane can result in cell death by disrupting essential intracellular processes, such as DNA transcription, ribosome function, and electron transport [171].

Another advantage of EOs is that they have little to no effect on the development of antimicrobial resistance, in contrast to traditional antibiotics [172]. In vitro studies have demonstrated the potential of EOs as novel treatments for multidrug-resistant microorganisms. For example, essential oils from thyme, peppermint, lavender, cinnamon, tea tree, rosemary, eucalyptus, and lemongrass have been found to possess antimicrobial properties [45]. In addition to their role as antibiotics and antiseptics, EOs have been incorporated into wound dressings to serve as effective antibacterial agents. However, despite their beneficial role in treating wound infections, repeated applications and/or high concentrations of essential oils may be necessary, which could potentially lead to adverse effects on the patient.

Nanostructures play a crucial role in the treatment of bacterial infections due to their high surface area and ability to encapsulate drugs. Nanoencapsulation of essential oils is an innovative approach that enhances their effectiveness in wound treatment systems by improving their antibacterial properties. This process increases the physical stability, bioactivity, and antibacterial potential of essential oils, while also reducing their volatility and toxicity [171]. Various methods can be employed to encapsulate essential oils, including polymer-based nanoparticles, nanocapsules, nanoemulsions, solid lipid nanocapsules, nanostructured lipid carriers, and liposomes. These techniques allow for better control of release behavior and enhanced antibacterial activity, making them promising solutions for wound care.

### 6.4. Iodine and Iodine Complexes

Iodine, a natural halogen, is a widely used antiseptic with various topical applications. It has a broad spectrum of activity, targeting bacteria, fungi, viruses, protozoa, and even prions through non-specific mechanisms [173]. Iodine has been employed to prevent and treat infections since the fourth century BCE, although its use has been the subject of ongoing debate [174]. This discussion gained prominence when Alexander Fleming recommended iodine as an antiseptic to prevent gas gangrene in wounds during World War I. While the use of antiseptics like iodine declined with the rise of antibiotic-resistant bacterial strains, modern iodine formulations continue to be explored for infection management, though results remain mixed, and no definitive consensus has been reached [175]. One of the most significant advancements in iodine formulations is the development of iodophors in the 1950s. These are created by complexing elemental iodine with a surfactant to improve solubility and reduce cytotoxicity. Elemental iodine is cytotoxic to fibroblasts, keratinocytes, and leukocytes, which impedes wound healing. Iodophors, such as povidone-iodine (PVP-I) and cadexomer-iodine, address this issue by releasing lower concentrations of free iodine into wound exudate, reducing the adverse effects on healthy tissue [102].

Povidone-iodine (PVP-I) is the most commonly used iodine formulation in clinical settings, but its long-term use, particularly in complex wounds, is not recommended. Research has shown that even clinical concentrations as low as 1% can be cytotoxic to granulocytes and monocytes in vitro, and PVP-I dressings, which typically contain up to 7.5% iodine, can lead to systemic iodine toxicity [176].

On the other hand, cadexomer-iodine formulations have shown promising results in controlling bacterial load in topical applications. Studies on human and porcine models have demonstrated that cadexomer-iodine accelerates epidermal migration and re-epithelialization by upregulating cytokines such as vascular endothelial growth factor (VEGF). In one small study, cadexomer-iodine was also shown to improve healing rates in chronic wounds [177]. Available in various forms such as ointments, powders, hydrogels, gauze, knitted viscose, beads, and pastes, cadexomer-iodine is a versatile option for wound care [178].

In summary, while iodine remains a key antiseptic in wound management, the formulations and concentrations used must be carefully considered to balance effectiveness and minimize cytotoxicity, with newer iodophor products like cadexomer-iodine showing potential benefits in specific wound healing applications.

### 6.5. Chlorhexidine Gluconate (CHG)

Antibacterial agents such as chlorhexidine have been incorporated into a wide range of commercially available dressings and washes [179]. Chlorhexidine gluconate (CHG), which has been used in infection control since the 1950s, works by binding to the cell walls of microorganisms. This binding disrupts the integrity of the cell membranes, causing leakage of cellular contents and ultimately leading to cell death. CHG is highly effective against a broad spectrum of microorganisms, including many bacteria, some fungi, and certain viruses, making it a versatile agent for infection control [46]. While generally effective, CHG can cause skin irritation or allergic reactions in some individuals, so it should be used with caution, particularly in sensitive populations [180].

Kapanya et al. [47] developed hydrogels incorporating CHG using poly(sodium 2-acrylamido-2-methylpropane sulfonate) and gelatin. Among the formulations, the hydrogel containing 0.02% *w*/*v* CHG was selected for further evaluation and compared against a drug-free control. The antibacterial efficacy of the hydrogels was assessed using the shake-flask method and scanning electron microscopy (SEM). Their results showed significant antibacterial activity with a 7-log reduction for *S. aureus* and a 5-6-log reduction for *E. coli*. Additionally, cytotoxicity was evaluated via an MTT assay, which demonstrated cell viability exceeding 70% after 24 h, indicating a non-cytotoxic profile. Overall, CHG-loaded hydrogels present a promising candidate for biomedical applications, particularly as antibacterial wound dressings.

Some CHG-incorporated dressings are designed for sustained release, ensuring prolonged antimicrobial activity. For example, CHG-loaded alginate fibers and polyurethane foam dressings maintain their efficacy over several days [48].

### 6.6. Silver

Silver has been regarded as an antimicrobial agent for thousands of years, before people knew about the word “microorganisms”. Silver Nitrate and Silver Sulfadiazine are traditional forms of silver that are widely used in clinical practice for their antimicrobial properties [181]. Thanks to the development of nanoscience and technology, nowadays silver is mainly applied in the form of nanoparticles [182]. Nanoparticles are increasingly being used in smart dressings due to their unique properties and ability to interact at the cellular level. They can be engineered to carry and deliver drugs, growth factors, or other bioactive compounds directly to the wound site. Some nanoparticles also exhibit antimicrobial properties, helping to prevent infections. Dressings with silver nanoparticles provide broad-spectrum antimicrobial activity and are effective against multidrug-resistant pathogens [49,183]. There have been many studies on the efficacy of silver ions and silver nanoparticles (AgNPs) against diverse bacterial pathogens [184,185]. AgNPs has been reported to have a better activity than Ag+, and their efficacy seems to be size-dependent, suggesting that AgNPs with a diameter of 1–10 nm can have a direct interaction with the bacteria [50].

Emerging trends in antimicrobial wound dressings are increasingly focused on advanced silver-based technologies, including nanocrystalline silver, for minimizing antibiotic resistance transmission and reducing systemic antibiotic use [24]. Biogenic silver nanoparticles, synthesized through biological methods, which offer reduced toxicity and effective antimicrobial action, are particularly beneficial for diabetic wound care [51]. Moreover, innovative hybrid nanoparticle designs, such as enzyme-core structures with porous metallic silver outer layers, are being explored to further enhance the efficacy of silver nanoparticles, promising a new generation of more potent and biocompatible wound treatments [186].

## 7. Challenges and Prospects in the Development of Smart Dressings

Personalized wound care is rapidly becoming the gold standard in managing complex wounds, recognizing that each patient presents a unique set of circumstances that influence healing. A “one-size-fits-all” approach often falls short, as factors like wound type, location, size, underlying health conditions (such as diabetes or vascular disease), age, and even lifestyle choices can significantly impact the healing trajectory. Personalized care takes these individual factors into account to create a tailored treatment plan that optimizes the healing process and improves patient outcomes. This approach moves beyond simply treating the wound and focuses on addressing the specific needs of the individual, leading to more efficient healing [187].

Smart dressings are at the forefront of enabling truly personalized wound care. Their inherent versatility allows them to be customized and adapted to meet the specific requirements of each patient. For instance, a patient with diabetes, who often experiences impaired wound healing due to reduced blood flow and nerve damage, might benefit from a smart dressing that incorporates sensors to monitor glucose levels in the wound bed and deliver growth factors to stimulate tissue regeneration. Similarly, a patient with a burn wound might require a dressing that can manage significant fluid loss and deliver antimicrobial agents to prevent infection. Smart dressings can also be tailored to address the specific needs of different wound types, such as pressure ulcers, venous leg ulcers, or surgical incisions, by incorporating appropriate materials, drug delivery systems, and sensing capabilities [188,189].

Beyond the specific needs of the wound itself, smart dressings can also be personalized based on patient-specific factors. For example, elderly patients with fragile skin might benefit from dressings made with gentle adhesives and materials that minimize trauma during dressing changes. Patients with limited mobility might benefit from dressings that require less frequent changes or can be monitored remotely, reducing the need for frequent clinic visits. Furthermore, the data collected by smart dressings can be used to track the healing progress over time and make adjustments to the treatment plan as needed. This real-time feedback loop allows healthcare providers to personalize the care even further, ensuring that the patient receives the most appropriate and effective treatment at every stage of the healing process. In essence, smart dressings empower healthcare providers to move away from reactive wound care to a proactive and personalized approach, leading to better outcomes and improved quality of life for patients.

The field of antimicrobial smart dressings is a dynamic area of research, with ongoing efforts focused on enhancing their efficacy, expanding their capabilities, and addressing existing limitations. One prominent area of investigation involves the development of novel antimicrobial agents that are less susceptible to resistance development. Researchers are exploring new classes of peptides, polymers, and nanomaterials with potent antimicrobial activity, aiming to create dressings that can effectively combat even drug-resistant pathogens. Furthermore, significant attention is being paid to the design of “smart” release mechanisms that can deliver these antimicrobials in a controlled and targeted manner. This includes stimuli-responsive dressings that release drugs based on changes in the wound environment, such as pH or temperature, as well as dressings that can be triggered externally, for example, by light or ultrasound. These controlled release systems aim to maximize the therapeutic effect while minimizing systemic toxicity and reducing the selective pressure for resistance.

Another exciting avenue of research focuses on integrating advanced sensing technologies into smart dressings. Scientists are working on miniaturizing and improving the sensitivity of biosensors that can detect a wide range of biomarkers in the wound microenvironment, including inflammatory markers, bacterial toxins, and growth factors. These sensors can provide real-time information about the wound’s condition, allowing healthcare providers to make informed decisions about treatment strategies. Moreover, efforts are underway to develop dressings that can not only sense but also actively respond to these changes, such as the presence detection of specific bacteria and automatically release the appropriate antimicrobial agent. This closed-loop feedback system represents a significant step toward truly intelligent wound care.

Emerging technologies are also playing a crucial role in the advancement of antimicrobial smart dressings. Nanotechnology, for example, is being used to create nanomaterials with enhanced antimicrobial properties and to develop nanoscale drug delivery systems that can penetrate biofilms and target specific microbial cells. Microfluidics is another promising technology that enables the precise manipulation of fluids at the microscale, allowing for the creation of dressings with complex architectures and integrated microchannels for drug delivery and sensing. Furthermore, additive manufacturing, or 3D printing, is emerging as a powerful tool for creating personalized smart dressings tailored to the unique shape and characteristics of individual wounds [190,191]. These 3D-printed dressings can be designed to incorporate multiple antimicrobial agents, sensors, and other therapeutic components, offering a highly customized and effective approach to wound care. As these and other emerging technologies continue to mature, we can expect to see a new generation of antimicrobial smart dressings that are more effective, more intelligent, and more personalized than ever before.

## 8. Conclusions

Looking ahead, the convergence of several key technological advancements promises to further revolutionize the field of smart dressings. The increasing sophistication of biosensors will allow for the detection of an even wider range of biomarkers, providing a more comprehensive picture of the wound microenvironment. The integration of artificial intelligence and machine learning algorithms will enable smart dressings to analyze these data and make autonomous decisions about drug delivery and other therapeutic interventions. This will pave the way for truly “self-regulating” dressings that can adapt to changing wound conditions without requiring frequent input from healthcare professionals. Moreover, the development of new biocompatible materials with inherent antimicrobial properties will reduce our reliance on traditional antibiotics, further mitigating the risk of resistance development. As these technologies mature and become more accessible, smart dressings will play an increasingly crucial role in combating antibiotic resistance, improving wound care outcomes, and ultimately, enhancing the quality of life for patients with acute and chronic wounds. This is directly linked to the continued development and refinement of smart dressings, particularly in the face of the growing global challenge of antibiotic resistance. The future of wound care is bright, and smart dressings are leading the charge.

As we move toward an era of personalized medicine, smart dressings offer a powerful platform for tailoring wound care strategies to the specific needs of each patient, optimizing healing outcomes and minimizing the selective pressure that drives resistance. The ability to monitor wound conditions in real time, deliver targeted therapies, and adapt treatment plans based on individual responses represents a paradigm shift from traditional passive wound care to a proactive and intelligent approach. By incorporating multiple antimicrobial strategies, such as novel agents, controlled release mechanisms, and biofilm-disrupting enzymes, smart dressings can provide a robust defense against infection, even in the presence of drug-resistant pathogens. Furthermore, the integration of advanced sensing technologies and data analytics will enable healthcare providers to gain a deeper understanding of the complex wound healing process, leading to more informed and effective interventions.

Future research efforts may focus on improving the delivery and efficacy of growth factors, designing biomaterials that closely mimic the extracellular matrix, and further elucidating the role of immune regulation in the wound healing process. The convergence of advanced technologies, including nanotechnology and 3D bioprinting, offers unprecedented opportunities to create next-generation, patient-specific wound dressings capable of both promoting tissue regeneration and mitigating the risk of resistant infections. By enabling controlled, stimuli-responsive antimicrobial release and fostering favorable wound microenvironments, these innovations can play a pivotal role in slowing the emergence of AMR in clinical settings. However, realizing this potential demands a coordinated, multidisciplinary effort, uniting materials science, microbiology, bioengineering, and clinical expertise. Only through such integrated approaches can we drive transformative progress in wound management, safeguard the efficacy of antimicrobial therapies, and ultimately improve long-term patient outcomes on a global scale.

Looking ahead, our research will prioritize the exploration of microneedle technology and magnetic materials for advanced wound care applications. Microneedle systems present a promising approach for the direct delivery of antimicrobial agents into the wound bed, facilitating higher local drug concentrations and enhancing therapeutic efficacy against pathogenic microorganisms. In parallel, magnetic materials, particularly magnetic nanoparticles (MNPs), offer innovative strategies to address antimicrobial resistance through mechanisms such as localized thermal inactivation, improved targeted drug delivery, and the disruption of biofilms. These materials can be incorporated into wound dressings and combined with complementary therapies to accelerate healing processes while simultaneously reducing the incidence of antibiotic-resistant infections. Together, all these technologies represent a significant step toward the development of next-generation, multifunctional wound management platforms.

## Figures and Tables

**Table 1:** Types of tests used for evaluating the smart dressings.

Method	Test	References
In vitro	Antimicrobial tests (on *Staphylococcus aureus, Pseudomonas aeruginosa, Escherichia coli*, and *Streptococcus pyogenes*)	[36,37,38,39,40,41,42,43,44,45,46,47,48,49,50,51]
	Sensor array operation in vitro in simulated infected wound models	[36]
	Test strip sensor for early and accurate detection of wound infection at the bedside	[52]
	Electrochemical behavior sensors	[53,54,55]
	Mechanical Test	[38,44,56]
	Dehydration and Hydration Tests	[38,44,48,56,57]
	Viability/Cytotoxicity Assay	[38,39,40,41,43,44,47,49,56]
	Biocompatibility test	[44,49,56]
	Drug release kinetics	[40,41,43,44,47]
	Antioxidant activity	[41]
	Detection of bacterial fluorescence	[42]
Ex-vivo	Biofilm formation on ex vivo porcine skin	[37]
	Ex Vivo Bacterial Detection Tests	[56]
In vivo	Animal model (bacterial inhibition, reduction angiogenesis, anti-inflammatory activity)	[38,39,42,43,44,51,53,58]

**Table 2:** Summary of modern wound dressings.

Type of Dressing	Actions	Application/Indications	Advantages	Desavantages	References
Films	Autolytic debridement	Superficial wounds with minimal exudate: minor burns, stage I and stage II pressure ulcers	Transparent-easy to visualize the wound Highly flexible Impermeable to liquid or bacteria	May adhere to wounds Not suitable for heavily draining wounds May promote macerations due to its occlusive nature No absorptive capacity	[27,79,80,81]
Foams	Absorb fluids Moisture control Flexible to the wound bed	Exudative wounds, deep wounds, burns, chronic wounds, lower leg ulcers	Existing low-adherent versions available for patients with fragile skin Great absorption	Not for use in dry or necrotic wounds	[82,83,84,85,86]
Hydrocolloids	Absorb fluids Promote autolytic debridement	Clean, low to moderate exuding wounds: Average thickness wounds, burns, chronic ulcers	Long wear-time Absorbent Occlusive Protects wound from contamination	May cause maceration	[87,88,89,90,91]
Hydrogels	Promote autolytic debridement Rehydrated wound bed Moisture control	Low to moderate exuding wound: Partial-thickness burns, dry chronic wounds, necrotic wounds, minor ulcerations, exposed bone	Provides moisture Easy removal Cooling effect	Expensive Biocompatibility issues May cause maceration	[7,92,93,94,95]
Nanofibers	Absorb exudate Promote autolytic debridement Moisture control	Surgical wounds, bleeding wounds, high-exudate chronic ulcers, sinus tracts	Excellent exudate absorbtion	Not for use in dry or necrotic wounds (can cause bleeding)	[67,96,97,98,99]

## Data Availability

No new data were created or analyzed in this study. Data sharing is not applicable to this article.

## Abbreviations

The following abbreviations are used in this manuscript:

BC	Before Christ
BCE	Before Christian Era
MRSA	Methicillin-resistant *Staphylococcus aureus*
MDR	Multidrug-resistant
PHMB	Polyhexamethylene biguanide
AMPs	Antimicrobial peptides
PTT	Photothermal therapy
PDT	Photodynamic therapy
ECM	Extracellular matrix
PLA	Polylactic acid
PLGA	Poly(lactic-*co*-glycolic acid)
PCL	Polycaprolactone
PVA	Polyvinyl alcohol
MPO	Myeloperoxidase
DNA	Deoxyribonucleic acid
E-DNA	Ultrasensitive electrochemical DNA
ISDPR	Isothermal strand-displacement polymerization reaction
CaP NPs	Calcium phosphate nanoparticles
TOB	Tobramycin
OD	Oxidized dextran
QCS	Quaternized chitosan
GelMA	Methacrylated gelatin
MUG	4-Methylumphulone beta-D-glucoside
CFU	Colony-forming unit
CuS NPs	Copper sulfide nanoparticles
UCNPs@TiO_2_	Up-converted nanoparticles coated with titanium dioxide
CIP	Ciprofloxacin
GS	Gentamicin sulfate
CMC	Carboxymethyl chitosan
COL	Collagen
EOs	Essential oils
PVP-I	Povidone-iodine
VEGF	Vascular endothelial growth factor
CHG	Chlorhexidine gluconate
SEM	Scanning electron microscopy
MTT	3-(4,5-Dimethylthiazol-2-yl)-2,5-diphenyltetrazolium bromide
AgNPs	Silver nanoparticles

## References

[1] Robson M.C., Steed D.L., Franz M.G. (2001). Wound Healing: Biologic Features and Approaches to Maximize Healing Trajectories. Curr. Probl. Surg..

[2] Las Heras K., Igartua M., Santos-Vizcaino E., Hernandez R.M. (2020). Chronic Wounds: Current Status, Available Strategies and Emerging Therapeutic Solutions. J. Control. Release.

[3] Eming S.A., Hammerschmidt M., Krieg T., Roers A. (2009). Interrelation of Immunity and Tissue Repair or Regeneration. Semin. Cell Dev. Biol..

[4] Fonder M.A., Lazarus G.S., Cowan D.A., Aronson-Cook B., Kohli A.R., Mamelak A.J. (2008). Treating the Chronic Wound: A Practical Approach to the Care of Nonhealing Wounds and Wound Care Dressings. J. Am. Acad. Dermatol..

[5] Han G., Ceilley R. (2017). Chronic Wound Healing: A Review of Current Management and Treatments. Adv. Ther..

[6] Thomas S., Uzun M. (2019). Testing Dressings and Wound Management Materials. Advanced Textiles for Wound Care.

[7] Brumberg V., Astrelina T., Malivanova T., Samoilov A. (2021). Modern Wound Dressings: Hydrogel Dressings. Biomedicines.

[8] Pranantyo D., Yeo C.K., Wu Y., Fan C., Xu X., Yip Y.S., Vos M.I.G., Mahadevegowda S.H., Lim P.L.K., Yang L. (2024). Hydrogel Dressings with Intrinsic Antibiofilm and Antioxidative Dual Functionalities Accelerate Infected Diabetic Wound Healing. Nat. Commun..

[9] Popovich K., Tohm P., Hurd T. (2010). Skin and Wound Care Excellence: Integrating Best-Practice Evidence. Healthc. Q.

[10] Collignon P. (2012). Clinical Impact of Antimicrobial Resistance in Humans. Rev. Sci. Tech. OIE.

[11] Yang C., Yang C., Chen Y., Liu J., Liu Z., Chen H.-J. (2023). The Trends in Wound Management: Sensing, Therapeutic Treatment, and “Theranostics”. J. Sci. Adv. Mater. Devices.

[12] Bowler P.G. (2018). Antibiotic Resistance and Biofilm Tolerance: A Combined Threat in the Treatment of Chronic Infections. J. Wound Care.

[13] Schultz G.S., Sibbald R.G., Falanga V., Ayello E.A., Dowsett C., Harding K., Romanelli M., Stacey M.C., Teot L., Vanscheidt W. (2003). Wound Bed Preparation: A Systematic Approach to Wound Management. Wound Repair Regen..

[14] Kirketerp-Møller K., Jensen P.Ø., Fazli M., Madsen K.G., Pedersen J., Moser C., Tolker-Nielsen T., Høiby N., Givskov M., Bjarnsholt T. (2008). Distribution, Organization, and Ecology of Bacteria in Chronic Wounds. J. Clin. Microbiol..

[15] Awuor S.O., Omwenga E.O., Mariita R.M., Musila J.M., Musyoki S. (2023). Monitoring the Battleground: Exploring Antimicrobial Resistance and Virulence Factors in Wound Bacterial Isolates. Access Microbiol..

[16] Howell-Jones R.S., Wilson M.J., Hill K.E., Howard A.J., Price P.E., Thomas D.W. (2005). A Review of the Microbiology, Antibiotic Usage and Resistance in Chronic Skin Wounds. J. Antimicrob. Chemother..

[17] Wolcott R.D., Hanson J.D., Rees E.J., Koenig L.D., Phillips C.D., Wolcott R.A., Cox S.B., White J.S. (2016). Analysis of the Chronic Wound Microbiota of 2,963 Patients by 16S rDNA Pyrosequencing. Wound Repair Regen..

[18] Smith D.M., Snow D.E., Rees E., Zischkau A.M., Hanson J.D., Wolcott R.D., Sun Y., White J., Kumar S., Dowd S.E. (2010). Evaluation of the Bacterial Diversity of Pressure Ulcers Using bTEFAP Pyrosequencing. BMC Med. Genom..

[19] Kolar M., Cermak P., Hobzova L., Bogdanova K., Neradova K., Mlynarcik P., Bostik P. (2020). Antibiotic Resistance in Nosocomial Bacteria Isolated from Infected Wounds of Hospitalized Patients in Czech Republic. Antibiotics.

[20] Filius P.M., Gyssens I.C. (2002). Impact of Increasing Antimicrobial Resistance on Wound Management. Am. J. Clin. Dermatol..

[21] Cassini A., Högberg L.D., Plachouras D., Quattrocchi A., Hoxha A., Simonsen G.S., Colomb-Cotinat M., Kretzschmar M.E., Devleesschauwer B., Cecchini M. (2019). Attributable Deaths and Disability-Adjusted Life-Years Caused by Infections with Antibiotic-Resistant Bacteria in the EU and the European Economic Area in 2015: A Population-Level Modelling Analysis. Lancet Infect. Dis..

[22] Edwards-Jones V. (2020). Antimicrobial Stewardship in Wound Care. Br. J. Nurs..

[23] Guest J.F., Ayoub N., McIlwraith T., Uchegbu I., Gerrish A., Weidlich D., Vowden K., Vowden P. (2017). Health Economic Burden That Different Wound Types Impose on the UK ’s National Health Service. Int. Wound J..

[24] Woodmansey E.J., Roberts C.D. (2018). Appropriate Use of Dressings Containing Nanocrystalline Silver to Support Antimicrobial Stewardship in Wounds. Int. Wound J..

[25] Chin J.D., Zhao L., Mayberry T.G., Cowan B.C., Wakefield M.R., Fang Y. (2023). Photodynamic Therapy, Probiotics, Acetic Acid, and Essential Oil in the Treatment of Chronic Wounds Infected with *Pseudomonas aeruginosa*. Pharmaceutics.

[26] Pang Q., Yang F., Jiang Z., Wu K., Hou R., Zhu Y. (2023). Smart Wound Dressing for Advanced Wound Management: Real-Time Monitoring and on-Demand Treatment. Mater. Des..

[27] Pirouzzadeh M., Moraffah F., Samadi N., Sharifzadeh M., Motasadizadeh H., Vatanara A. (2025). Enhancement of Burn Wound Healing Using Optimized Bioactive Probiotic-Loaded Alginate Films. Int. J. Biol. Macromol..

[28] Wolcott R. (2016). Are Chronic Wounds, Chronic Infections?. J. Wound Care.

[29] Hurlow J., Bowler P.G. (2022). Acute and Chronic Wound Infections: Microbiological, Immunological, Clinical and Therapeutic Distinctions. J. Wound Care.

[30] Percival S.L., McCarty S.M., Lipsky B. (2015). Biofilms and Wounds: An Overview of the Evidence. Adv. Wound Care.

[31] Sen C.K., Roy S., Mathew-Steiner S.S., Gordillo G.M. (2021). Biofilm Management in Wound Care. Plast. Reconstr. Surg..

[32] Zhao G., Usui M.L., Lippman S.I., James G.A., Stewart P.S., Fleckman P., Olerud J.E. (2013). Biofilms and Inflammation in Chronic Wounds. Adv. Wound Care.

[33] Watters C., Fleming D., Bishop D., Rumbaugh K.P. (2016). Host Responses to Biofilm. Progress in Molecular Biology and Translational Science.

[34] Verderosa A.D., Totsika M., Fairfull-Smith K.E. (2019). Bacterial Biofilm Eradication Agents: A Current Review. Front. Chem..

[35] Huemer M., Mairpady Shambat S., Brugger S.D., Zinkernagel A.S. (2020). Antibiotic Resistance and Persistence—Implications for Human Health and Treatment Perspectives. EMBO Rep..

[36] Sheybani R., Shukla A. (2017). Highly Sensitive Label-Free Dual Sensor Array for Rapid Detection of Wound Bacteria. Biosens. Bioelectron..

[37] Magee E., Yusufu D., Rice C.J., Skvortsov T., Mills A., Gilmore B.F. (2024). A Smart Sensor for Monitoring Antimicrobial Interventions in Wound Infections. Sens. Actuators B Chem..

[38] Fang H., Wang J., Li L., Xu L., Wu Y., Wang Y., Fei X., Tian J., Li Y. (2019). A Novel High-Strength Poly(ionic liquid)/PVA Hydrogel Dressing for Antibacterial Applications. Chem. Eng. J..

[39] Zhou J., Yao D., Qian Z., Hou S., Li L., Jenkins A.T.A., Fan Y. (2018). Bacteria-Responsive Intelligent Wound Dressing: Simultaneous In Situ Detection and Inhibition of Bacterial Infection for Accelerated Wound Healing. Biomaterials.

[40] Huang T., Sun Z., Heath D.E., O’Brien-Simpson N., O’Connor A.J. (2024). 3D Printed and Smart Alginate Wound Dressings with pH-Responsive Drug and Nanoparticle Release. Chem. Eng. J..

[41] Huang Y., Mu L., Zhao X., Han Y., Guo B. (2022). Bacterial Growth-Induced Tobramycin Smart Release Self-Healing Hydrogel for *Pseudomonas aeruginosa*-Infected Burn Wound Healing. ACS Nano.

[42] Yang J., He Y., Li Z., Yang X., Gao Y., Chen M., Zheng Y., Mao S., Shi X. (2024). Intelligent Wound Dressing for Simultaneous in Situ Detection and Elimination of Pathogenic Bacteria. Acta Biomater..

[43] Shi Y., Zhou M., Zhao S., Li H., Wang W., Cheng J., Jin L., Wang Y. (2023). Janus Amphiphilic Nanofiber Membranes Synergistically Drive Antibacterial and Anti-Inflammatory Strategies for Skin Wound Healing. Mater. Des..

[44] Hu S., Cai X., Qu X., Yu B., Yan C., Yang J., Li F., Zheng Y., Shi X. (2019). Preparation of Biocompatible Wound Dressings with Long-Term Antimicrobial Activity through Covalent Bonding of Antibiotic Agents to Natural Polymers. Int. J. Biol. Macromol..

[45] Zenati F., Benbelaid F., Khadir A., Bellahsene C., Bendahou M. (2014). Antimicrobial Effects of Three Essential Oils on Multidrug Resistant Bacteria Responsible for Urinary Infections. J. App. Pharm. Sci..

[46] Cheung H.-Y., Wong M.M.-K., Cheung S.-H., Liang L.Y., Lam Y.-W., Chiu S.-K. (2012). Differential Actions of Chlorhexidine on the Cell Wall of *Bacillus subtilis* and *Escherichia coli*. PLoS ONE.

[47] Kapanya A., Somsunan R., Molloy R., Jiranusornkul S., Leewattanapasuk W., Jongpaiboonkit L., Kong Y. (2020). Synthesis of Polymeric Hydrogels Incorporating Chlorhexidine Gluconate as Antibacterial Wound Dressings. J. Biomater. Sci. Polym. Ed..

[48] Shi S.-S., Lei S.-J., Fu C. (2020). Studies of the Properties of CHG-Loaded Alginate Fibers for Medical Application. Polym. Test..

[49] Składanowski M., Golinska P., Rudnicka K., Dahm H., Rai M. (2016). Evaluation of Cytotoxicity, Immune Compatibility and Antibacterial Activity of Biogenic Silver Nanoparticles. Med. Microbiol. Immunol..

[50] Finley P.J., Norton R., Austin C., Mitchell A., Zank S., Durham P. (2015). Unprecedented Silver Resistance in Clinically Isolated Enterobacteriaceae: Major Implications for Burn and Wound Management. Antimicrob. Agents Chemother..

[51] Krishnan N., Velramar B., Ramatchandirin B., Abraham G.C., Duraisamy N., Pandiyan R., Velu R.K. (2018). Effect of Biogenic Silver Nanocubes on Matrix Metalloproteinases 2 and 9 Expressions in Hyperglycemic Skin Injury and Its Impact in Early Wound Healing in Streptozotocin-Induced Diabetic Mice. Mater. Sci. Eng. C.

[52] Prader J., Rumpler M., Kamolz L.P., Hajnsek M. (2022). Myeloperoxidase-Based in-Vitro Test Strip Sensor for Early Detection of Wound Infections at the Patient’s Bedside. Sens. Actuators B Chem..

[53] Thirabowonkitphithan P., Phuengmaung P., Leelahavanichkul A., Laiwattanapaisal W. (2023). MWCNTs/PVA Hydrogel-Modified Electrochemical Sensors for Ex Vivo and In Vivo Detection of Pyocyanin Biomarker for *Pseudomonas aeruginosa* Wound Infection. ACS Appl. Electron. Mater..

[54] Sismaet H.J., Banerjee A., McNish S., Choi Y., Torralba M., Lucas S., Chan A., Shanmugam V.K., Goluch E.D. (2016). Electrochemical Detection of *Pseudomonas* in Wound Exudate Samples from Patients with Chronic Wounds. Wound Repair Regen..

[55] Wang T., Zhang Z., Li Y., Xie G. (2015). Amplified Electrochemical Detection of mecA Gene in Methicillin-Resistant *Staphylococcus aureus* Based on Target Recycling Amplification and Isothermal Strand-Displacement Polymerization Reaction. Sens. Actuators B Chem..

[56] Mirani B., Pagan E., Currie B., Siddiqui M.A., Hosseinzadeh R., Mostafalu P., Zhang Y.S., Ghahary A., Akbari M. (2017). An Advanced Multifunctional Hydrogel-Based Dressing for Wound Monitoring and Drug Delivery. Adv. Healthc. Mater..

[57] Sta. Agueda J.R., Lim J., Mondragon J.M., Madrid J., Belen M.G., Eustaquio G.M.Y., Monjardin J.G., Salud N. (2021). Rapid Prototyping of a Temperature, Humidity, and Pressure Monitor Electronic Layer for Pressure Ulcer Wound Patch. J. Phys. Conf. Ser..

[58] Hickle K., Slamin R., Baez A., Sen D., Evan-Browning E., Tessier H., Mendelson Y., McNeill J., Dunn R. (2019). Wireless Pressure Ulcer Sensor: Validation in an Animal Model. Ann. Plast. Surg..

[59] Boateng J.S., Matthews K.H., Stevens H.N.E., Eccleston G.M. (2008). Wound Healing Dressings and Drug Delivery Systems: A Review. J. Pharm. Sci..

[60] Farahani M., Shafiee A. (2021). Wound Healing: From Passive to Smart Dressings. Adv. Healthc. Mater..

[61] Borda L.J., Macquhae F.E., Kirsner R.S. (2016). Wound Dressings: A Comprehensive Review. Curr. Derm. Rep..

[62] Ghanim Z., Alkotaji M., Qazzaz M. (2023). Insight into Topical Preparations for Wound Healing: Traditional and Modern Dressings. Al-Anbar Med. J. AMJ.

[63] Gao Y., Elhadad A., Choi S. (2024). Janus Paper-Based Wound Dressings for Effective Exudate Absorption and Antibiotic Delivery. Adv. Eng. Mater..

[64] Slater M. (2008). Does Moist Wound Healing Influence the Rate of Infection?. Br. J. Nurs..

[65] Wang X., Chen C., Hu J., Liu C., Ning Y., Lu F. (2024). Current Strategies for Monitoring and Controlling Bacterial Biofilm Formation on Medical Surfaces. Ecotoxicol. Environ. Saf..

[66] Bowler P.G., Duerden B.I., Armstrong D.G. (2001). Wound Microbiology and Associated Approaches to Wound Management. Clin. Microbiol. Rev..

[67] Lin P., He K., Luo J., Wang J., Liu Y., Zhang J., Fan H., Huang S., Lan W., Wang W. (2025). Multifunctional Nanofiber System with Photothermal-Controlled Drug Delivery and Motion Monitoring Capabilities as Intelligent Wound Dressing. Chem. Eng. J..

[68] Tottoli E.M., Dorati R., Genta I., Chiesa E., Pisani S., Conti B. (2020). Skin Wound Healing Process and New Emerging Technologies for Skin Wound Care and Regeneration. Pharmaceutics.

[69] Hall C.W., Mah T.-F. (2017). Molecular Mechanisms of Biofilm-Based Antibiotic Resistance and Tolerance in Pathogenic Bacteria. FEMS Microbiol. Rev..

[70] Ventola C.L. (2015). The Antibiotic Resistance Crisis: Part 1: Causes and Threats. Pharm. Ther..

[71] Jones V., Grey J.E., Harding K.G. (2006). Wound Dressings. BMJ.

[72] Li F., Qin Y., Lee J., Liao H., Wang N., Davis T.P., Qiao R., Ling D. (2020). Stimuli-Responsive Nano-Assemblies for Remotely Controlled Drug Delivery. J. Control. Release.

[73] Jia X., Dou Z., Zhang Y., Li F., Xing B., Hu Z., Li X., Liu Z., Yang W., Liu Z. (2023). Smart Responsive and Controlled-Release Hydrogels for Chronic Wound Treatment. Pharmaceutics.

[74] Ahmed S., Ahmad M., Swami B.L., Ikram S. (2016). A Review on Plants Extract Mediated Synthesis of Silver Nanoparticles for Antimicrobial Applications: A Green Expertise. J. Adv. Res..

[75] Rai M.K., Deshmukh S.D., Ingle A.P., Gade A.K. (2012). Silver Nanoparticles: The Powerful Nanoweapon against Multidrug-Resistant Bacteria: Activity of Silver Nanoparticles against MDR Bacteria. J. Appl. Microbiol..

[76] Patra J.K., Das G., Fraceto L.F., Campos E.V.R., Rodriguez-Torres M.D.P., Acosta-Torres L.S., Diaz-Torres L.A., Grillo R., Swamy M.K., Sharma S. (2018). Nano Based Drug Delivery Systems: Recent Developments and Future Prospects. J. Nanobiotechnol..

[77] Cedillo-Cortezano M., Martinez-Cuevas L.R., López J.A.M., Barrera López I.L., Escutia-Perez S., Petricevich V.L. (2024). Use of Medicinal Plants in the Process of Wound Healing: A Literature Review. Pharmaceuticals.

[78] Majtan J. (2014). Honey: An Immunomodulator in Wound Healing. Wound Repair Regen..

[79] Meuleneire F. (2014). A Vapour-Permeable Film Dressing Used on Superficial Wounds. Br. J. Nurs..

[80] Ottaviano L., Buoso S., Zamboni R., Sotgiu G., Posati T. (2025). Natural Protein Films from Textile Waste for Wound Healing and Wound Dressing Applications. J. Funct. Biomater..

[81] Li X., Yang X., Wang Z., Liu Y., Guo J., Zhu Y., Shao J., Li J., Wang L., Wang K. (2022). Antibacterial, Antioxidant and Biocompatible Nanosized Quercetin-PVA Xerogel Films for Wound Dressing. Colloids Surf. B Biointerfaces.

[82] Kim W.I., Ko Y.-G., Park M.R., Jung K.H., Kwon O.H. (2018). Preparation and Characterization of Polyurethane Foam Dressings Containing Natural Antimicrobial Agents for Wound Healing. Polymer.

[83] Li J., Xu X., Ma X., Cui M., Wang X., Chen J., Zhu J., Chen J. (2024). Antimicrobial Nonisocyanate Polyurethane Foam Derived from Lignin for Wound Healing. ACS Appl. Bio Mater..

[84] Chaganti P., Gordon I., Chao J.H., Zehtabchi S. (2019). A Systematic Review of Foam Dressings for Partial Thickness Burns. Am. J. Emerg. Med..

[85] Li S., Zhang Y., Ma X., Qiu S., Chen J., Lu G., Jia Z., Zhu J., Yang Q., Chen J. (2022). Antimicrobial Lignin-Based Polyurethane/Ag Composite Foams for Improving Wound Healing. Biomacromolecules.

[86] Boyar V. (2021). Successful Management of Complex Pediatric and Neonatal Wounds With Methylene Blue and Gentian Violet Foam Dressings. Wounds.

[87] Kamińska M.S., Cybulska A.M., Skonieczna-Żydecka K., Augustyniuk K., Grochans E., Karakiewicz B. (2020). Effectiveness of Hydrocolloid Dressings for Treating Pressure Ulcers in Adult Patients: A Systematic Review and Meta-Analysis. Int. J. Environ. Res. Public Health.

[88] Lee O.J., Kim J.-H., Moon B.M., Chao J.R., Yoon J., Ju H.W., Lee J.M., Park H.J., Kim D.W., Kim S.J. (2016). Fabrication and Characterization of Hydrocolloid Dressing with Silk Fibroin Nanoparticles for Wound Healing. Tissue Eng. Regen. Med..

[89] Jafari D., Moosazadeh Moghaddam M., Fallah Tafti M., Mirnejad R. (2025). Fabrication of an Antibacterial Hydrocolloid Dressing for the Management of Wound Infection Caused by Antibiotic-Resistant Bacteria: In Vitro Study. Mater. Today Commun..

[90] Yanagibayashi S., Kishimoto S., Ishihara M., Murakami K., Aoki H., Takikawa M., Fujita M., Sekido M., Kiyosawa T. (2012). Novel Hydrocolloid-Sheet as Wound Dressing to Stimulate Healing-Impaired Wound Healing in Diabetic Db/Db Mice. Bio-Med. Mater. Eng..

[91] Abraham S., Harsha G.G.S., Desai K., Furtado S., Srinivasan B. (2022). Nano Calcium Oxide Incorporated Hydrocolloid Dressings for Wound Care. J. Pharm. Innov..

[92] Vasile C., Pamfil D., Stoleru E., Baican M. (2020). New Developments in Medical Applications of Hybrid Hydrogels Containing Natural Polymers. Molecules.

[93] Bashir S., Hina M., Iqbal J., Rajpar A.H., Mujtaba M.A., Alghamdi N.A., Wageh S., Ramesh K., Ramesh S. (2020). Fundamental Concepts of Hydrogels: Synthesis, Properties, and Their Applications. Polymers.

[94] Liu L., Feng X., Pei Y., Wang J., Ding J., Chen L. (2018). α-Cyclodextrin Concentration-Controlled Thermo-Sensitive Supramolecular Hydrogels. Mater. Sci. Eng. C.

[95] Li H., Yang J., Hu X., Liang J., Fan Y., Zhang X. (2011). Superabsorbent Polysaccharide Hydrogels Based on Pullulan Derivate as Antibacterial Release Wound Dressing. J. Biomed. Mater. Res..

[96] Keshvardoostchokami M., Majidi S.S., Huo P., Ramachandran R., Chen M., Liu B. (2020). Electrospun Nanofibers of Natural and Synthetic Polymers as Artificial Extracellular Matrix for Tissue Engineering. Nanomaterials.

[97] AL-Jbour N.D., Beg M.D., Gimbun J., Alam A.K.M.M. (2019). An Overview of Chitosan Nanofibers and Their Applications in the Drug Delivery Process. Curr. Drug Deliv..

[98] Jayakumar A., Radoor S., Kim J.T., Rhim J.W., Parameswaranpillai J., Siengchin S. (2023). Functionalized Nanofiber for Wound Healing and Wound Dressing Applications. Functionalized Nanofibers.

[99] Zhou Z., Li C., Zeng Y., Huang T., Jiang X., Yu D.-G., Wang K. (2024). Natural Polymer Nanofiber Dressings for Effective Management of Chronic Diabetic Wounds: A Comprehensive Review. Int. J. Biol. Macromol..

[100] Kannon G.A., Garrett A.B. (1995). Moist Wound Healing with Occlusive Dressings: A Clinical Review. Dermatol. Surg..

[101] Firlar I., Altunbek M., McCarthy C., Ramalingam M., Camci-Unal G. (2022). Functional Hydrogels for Treatment of Chronic Wounds. Gels.

[102] Jones A.M., San Miguel L. (2006). Are Modern Wound Dressings a Clinical and Cost-Effective Alternative to the Use of Gauze?. J. Wound Care.

[103] Thomas S. (2008). Hydrocolloid Dressings in the Management of Acute Wounds: A Review of the Literature. Int. Wound J..

[104] Nguyen N., Dulai A.S., Adnan S., Khan Z., Sivamani R.K. (2025). Narrative Review of the Use of Hydrocolloids in Dermatology: Applications and Benefits. J. Clin. Med..

[105] Sibbald R.G., Coutts P., Woo K.Y. (2011). Reduction of Bacterial Burden and Pain in Chronic Wounds Using a New Polyhexamethylene Biguanide Antimicrobial Foam Dressing-Clinical Trial Results. Adv. Ski. Wound Care.

[106] Chaiarwut S., Choipang C., Sangsanoh P., Niyompanich J., Supaphol P. (2023). Using Natural Extracts to Promote the Antibacterial and Anti-Inflammatory Performance of Polyurethane Foams. J. Polym. Environ..

[107] Hunter A.M., Grigson C., Wade A. (2018). Influence of Topically Applied Menthol Cooling Gel on Soft Tissue Thermodynamics and Arterial and Cutaneous Blood Flow at Rest. Int. J. Sports Phys. Ther..

[108] Ng V.W.L., Chan J.M.W., Sardon H., Ono R.J., García J.M., Yang Y.Y., Hedrick J.L. (2014). Antimicrobial Hydrogels: A New Weapon in the Arsenal against Multidrug-Resistant Infections. Adv. Drug Deliv. Rev..

[109] Liu J., Jiang W., Xu Q., Zheng Y. (2022). Progress in Antibacterial Hydrogel Dressing. Gels.

[110] Wang X., Zhao D., Li Y., Zhou X., Hui Z., Lei X., Qiu L., Bai Y., Wang C., Xia J. (2023). Collagen Hydrogel with Multiple Antimicrobial Mechanisms as Anti-Bacterial Wound Dressing. Int. J. Biol. Macromol..

[111] Jiang S., Deng J., Jin Y., Qian B., Lv W., Zhou Q., Mei E., Neisiany R.E., Liu Y., You Z. (2023). Breathable, Antifreezing, Mechanically Skin-like Hydrogel Textile Wound Dressings with Dual Antibacterial Mechanisms. Bioact. Mater..

[112] Liu X., Xu H., Zhang M., Yu D.-G. (2021). Electrospun Medicated Nanofibers for Wound Healing: Review. Membranes.

[113] Clark M., Rehm B.H.A., Moradali M.F. (2018). Alginates in Dressings and Wound Management. Alginates and Their Biomedical Applications.

[114] Berechet M.D., Gaidau C., Miletic A., Pilic B., Râpă M., Stanca M., Ditu L.-M., Constantinescu R., Lazea-Stoyanova A. (2020). Bioactive Properties of Nanofibres Based on Concentrated Collagen Hydrolysate Loaded with Thyme and Oregano Essential Oils. Materials.

[115] Chen H., Shen Y., Zhang H., Long X., Deng K., Xu T., Li Y. (2022). Clinical Application of Polylactic Acid/Gelatin Nanofibre Membrane in Hard-to-Heal Lower Extremity Venous Ulcers. J. Wound Care.

[116] Yukseloglu S.M., Sokmen N., Canoglu S. (2015). Biomaterial Applications of Silk Fibroin Electrospun Nanofibres. Microelectron. Eng..

[117] Sahoo R., Dash B.P., Panda P.K. (2023). Polyacrylonitrile and Polylactic Acid Blend Nanofibre Spinning Using Needleless Electrospinning Technique. Indian J. Fibre Text. Res. (IJFTR).

[118] Ma W., Zhou M., Dong W., Zhao S., Wang Y., Yao J., Liu Z., Han H., Sun D., Zhang M. (2021). A Bi-Layered Scaffold of a Poly(lactic-*co*-glycolic acid) Nanofiber Mat and an Alginate–Gelatin Hydrogel for Wound Healing. J. Mater. Chem. B.

[119] Jirkovec R., Erben J., Samkova A., Chaloupek J., Chvojka J. (2022). The Effect of the Electrospinning Setup on the Surface Energy of Polycaprolactone Nanofibre Layers. J. Ind. Text..

[120] Fertah M., Belfkira A., Dahmane E.M., Taourirte M., Brouillette F. (2017). Extraction and Characterization of Sodium Alginate from Moroccan *Laminaria digitata* Brown Seaweed. Arab. J. Chem..

[121] Yang C.H., Wang M.X., Haider H., Yang J.H., Sun J.-Y., Chen Y.M., Zhou J., Suo Z. (2013). Strengthening Alginate/Polyacrylamide Hydrogels Using Various Multivalent Cations. ACS Appl. Mater. Interfaces.

[122] Topuz F., Henke A., Richtering W., Groll J. (2012). Magnesium Ions and Alginate Do Form Hydrogels: A Rheological Study. Soft Matter.

[123] Aderibigbe B., Buyana B. (2018). Alginate in Wound Dressings. Pharmaceutics.

[124] Homaeigohar S., Boccaccini A.R. (2020). Antibacterial Biohybrid Nanofibers for Wound Dressings. Acta Biomater..

[125] Lu X., Zhou L., Song W. (2024). Recent Progress of Electrospun Nanofiber Dressing in the Promotion of Wound Healing. Polymers.

[126] Hodaei H., Esmaeili Z., Erfani Y., Esnaashari S.S., Geravand M., Adabi M. (2024). Preparation of Biocompatible Zein/Gelatin/Chitosan/PVA Based Nanofibers Loaded with Vitamin E-TPGS via Dual-Opposite Electrospinning Method. Sci. Rep..

[127] Xue J., Wu T., Dai Y., Xia Y. (2019). Electrospinning and Electrospun Nanofibers: Methods, Materials, and Applications. Chem. Rev..

[128] Durán N., Durán M., De Jesus M.B., Seabra A.B., Fávaro W.J., Nakazato G. (2016). Silver Nanoparticles: A New View on Mechanistic Aspects on Antimicrobial Activity. Nanomed. Nanotechnol. Biol. Med..

[129] Woodruff M.A., Hutmacher D.W. (2010). The Return of a Forgotten Polymer—Polycaprolactone in the 21st Century. Prog. Polym. Sci..

[130] Sill T.J., Von Recum H.A. (2008). Electrospinning: Applications in Drug Delivery and Tissue Engineering. Biomaterials.

[131] Ousey K., Rippon M.G., Rogers A.A., Totty J.P. (2023). Considerations for an Ideal Post-Surgical Wound Dressing Aligned with Antimicrobial Stewardship Objectives: A Scoping Review. J. Wound Care.

[132] Bagherabadi M., Feuilloley C., Cameron P.J., Andrieu-Brunsen A. (2025). Simultaneous Bacteria Sensing and On-Demand Antimicrobial Peptide Release. ACS Appl. Bio Mater..

[133] Derakhshandeh H., Aghabaglou F., McCarthy A., Mostafavi A., Wiseman C., Bonick Z., Ghanavati I., Harris S., Kreikemeier-Bower C., Moosavi Basri S.M. (2020). A Wirelessly Controlled Smart Bandage with 3D-Printed Miniaturized Needle Arrays. Adv. Funct. Mater..

[134] Tang N., Zheng Y., Cui D., Haick H. (2021). Multifunctional Dressing for Wound Diagnosis and Rehabilitation. Adv. Healthc. Mater..

[135] Bal-Öztürk A., Özkahraman B., Özbaş Z., Yaşayan G., Tamahkar E., Alarçin E. (2021). Advancements and Future Directions in the Antibacterial Wound Dressings—A Review. J. Biomed. Mater. Res..

[136] Pusta A., Tertiș M., Cristea C., Mirel S. (2021). Wearable Sensors for the Detection of Biomarkers for Wound Infection. Biosensors.

[137] Mota F.A.R., Pereira S.A.P., Araújo A.R.T.S., Passos M.L.C., Saraiva M.L.M.F.S. (2021). Biomarkers in the Diagnosis of Wounds Infection: An Analytical Perspective. TrAC Trends Anal. Chem..

[138] Li J., Li Z., Xiao J., Nie C. (2023). Conformable Electrochemical Devices for Closed-Loop Wound Management. Front. Bioeng. Biotechnol..

[139] Chen Z., Dong Y., Fu J., Bai Y., Gao Q., Qin Z., Wang J., Li S. (2024). Collaborative Biofluid Analysis Based Multi-Channel Integrated Wearable Detection System for the Monitoring of Wound Infection. Biosens. Bioelectron. X.

[140] Permpoka K., Purinai P., Cheerasiri C., Rojpalakorn W., Nilaratanakul V., Laiwattanapaisal W. (2024). Smartphone-Enabled 3D Origami-Fluidic Paper-Based Electrochemical Detection of Myeloperoxidase Activity for Assessing Wound Infection. Sens. Actuators B Chem..

[141] Sun X., Zhang Y., Ma C., Yuan Q., Wang X., Wan H., Wang P. (2021). A Review of Recent Advances in Flexible Wearable Sensors for Wound Detection Based on Optical and Electrical Sensing. Biosensors.

[142] Landsman A.S., Barnhart D., Sowa M. (2018). Near-Infrared Spectroscopy Imaging for Assessing Skin and Wound Oxygen Perfusion. Clin. Podiatr. Med. Surg..

[143] Li Q., Qi P., Wang Y., Fu S., Zhang H., Li S., Wang L., He C., Chen S., Hou P. (2024). Rapid-Response near-Infrared Fluorescence Probe for Colorimetric Detection of HClO and Its Applications in Environmental Monitoring and Biological Imaging. Spectrochim. Acta Part A Mol. Biomol. Spectrosc..

[144] Rasheed S., Kanwal T., Ahmad N., Fatima B., Najam-ul-Haq M., Hussain D. (2024). Advances and Challenges in Portable Optical Biosensors for Onsite Detection and Point-of-Care Diagnostics. TrAC Trends Anal. Chem..

[145] Casey V., McAree B., Moloney M.C., Grace P. (2010). Wearable Sub-Bandage Pressure Measurement System. Proceedings of the 2010 IEEE Sensors Applications Symposium (SAS).

[146] Dargaville T.R., Farrugia B.L., Broadbent J.A., Pace S., Upton Z., Voelcker N.H. (2013). Sensors and Imaging for Wound Healing: A Review. Biosens. Bioelectron..

[147] Xiao Y., Lai R.Y., Plaxco K.W. (2007). Preparation of Electrode-Immobilized, Redox-Modified Oligonucleotides for Electrochemical DNA and Aptamer-Based Sensing. Nat. Protoc..

[148] Saghazadeh S., Rinoldi C., Schot M., Kashaf S.S., Sharifi F., Jalilian E., Nuutila K., Giatsidis G., Mostafalu P., Derakhshandeh H. (2018). Drug Delivery Systems and Materials for Wound Healing Applications. Adv. Drug Deliv. Rev..

[149] Dong R., Guo B. (2021). Smart Wound Dressings for Wound Healing. Nano Today.

[150] Butcher M. (2011). Introducing a New Paradigm for Bioburden Management. J. Wound Care.

[151] Rippon M.G., Rogers A.A., Ousey K. (2021). Estrategias de Protección Antimicrobiana En El Cuidado de Heridas: Evidencia Para El Uso de Apósitos Recubiertos Con DACC. J. Wound Care.

[152] Zhang W., Hu X., Jiang F., Li Y., Chen W., Zhou T. (2024). Preparation of Bacterial Cellulose/Acrylic Acid-Based pH-Responsive Smart Dressings by Graft Copolymerization Method. J. Biomater. Sci. Polym. Ed..

[153] Deng P., Shi Z., Fang F., Xu Y., Zhou L., Liu Y., Jin M., Chen T., Wang Y., Cao Y. (2025). Wireless Matrix Metalloproteinase-9 Sensing by Smart Wound Dressing with Controlled Antibacterial Nanoparticles Release toward Chronic Wound Management. Biosens. Bioelectron..

[154] Wang X., Zhong B., Lou Z., Han W., Wang L. (2024). The Advancement of Intelligent Dressings for Monitoring Chronic Wound Infections. Chem. Eng. J..

[155] Echague C.G., Hair P.S., Cunnion K.M. (2010). A Comparison of Antibacterial Activity against Methicillin-Resistant *Staphylococcus aureus* and Gram-Negative Organisms for Antimicrobial Compounds in a Unique Composite Wound Dressing. Adv. Ski. Wound Care.

[156] Ilyas F., James A., Khan S., Haider S., Ullah S., Darwish G., Taqvi S.A.H.R., Ali R., Younas Q., Rehman A. (2024). Multidrug-Resistant Pathogens in Wound Infections: A Systematic Review. Cureus.

[157] Falcone M., De Angelis B., Pea F., Scalise A., Stefani S., Tasinato R., Zanetti O., Dalla Paola L. (2021). Challenges in the Management of Chronic Wound Infections. J. Glob. Antimicrob. Resist..

[158] Pogue J.M., Kaye K.S., Cohen D.A., Marchaim D. (2015). Appropriate Antimicrobial Therapy in the Era of Multidrug-Resistant Human Pathogens. Clin. Microbiol. Infect..

[159] Hemmati J., Azizi M., Asghari B., Arabestani M.R. (2023). Multidrug-Resistant Pathogens in Burn Wound, Prevention, Diagnosis, and Therapeutic Approaches (Conventional Antimicrobials and Nanoparticles). Can. J. Infect. Dis. Med. Microbiol..

[160] Helmy Y.A., Taha-Abdelaziz K., Hawwas H.A.E.-H., Ghosh S., AlKafaas S.S., Moawad M.M.M., Saied E.M., Kassem I.I., Mawad A.M.M. (2023). Antimicrobial Resistance and Recent Alternatives to Antibiotics for the Control of Bacterial Pathogens with an Emphasis on Foodborne Pathogens. Antibiotics.

[161] Shrestha G., Raphael J., Leavitt S.D., St. Clair L.L. (2014). In Vitro Evaluation of the Antibacterial Activity of Extracts from 34 Species of North American Lichens. Pharm. Biol..

[162] Molan P.C. (2006). The Evidence Supporting the Use of Honey as a Wound Dressing. Int. J. Low Extrem. Wounds.

[163] Jull A.B., Rodgers A., Walker N. (2008). Honey as a Topical Treatment for Wounds. Cochrane Database Syst. Rev..

[164] Cooper R. (2008). Using Honey to Inhibit Wound Pathogens. Nurs. Times.

[165] McLoone P., Oluwadun A., Warnock M., Fyfe L. (2016). Honey: A Therapeutic Agent for Disorders of the Skin. Cent. Asian J. Glob. Health.

[166] Cooper R. (2007). Honey in Wound Care: Antibacterial Properties. GMS Krankenhaushygiene Interdiszip..

[167] Seow Y.X., Yeo C.R., Chung H.L., Yuk H.-G. (2014). Plant Essential Oils as Active Antimicrobial Agents. Crit. Rev. Food Sci. Nutr..

[168] Semeniuc C.A., Pop C.R., Rotar A.M. (2017). Antibacterial Activity and Interactions of Plant Essential Oil Combinations against Gram-Positive and Gram-Negative Bacteria. J. Food Drug Anal..

[169] Aumeeruddy-Elalfi Z., Gurib-Fakim A., Mahomoodally M.F. (2016). Chemical Composition, Antimicrobial and Antibiotic Potentiating Activity of Essential Oils from 10 Tropical Medicinal Plants from Mauritius. J. Herb. Med..

[170] Kavoosi G., Tafsiry A., Ebdam A.A., Rowshan V. (2013). Evaluation of Antioxidant and Antimicrobial Activities of Essential Oils from *Carum copticum* Seed and *Ferula assafoetida* Latex. J. Food Sci..

[171] Rai M., Paralikar P., Jogee P., Agarkar G., Ingle A.P., Derita M., Zacchino S. (2017). Synergistic Antimicrobial Potential of Essential Oils in Combination with Nanoparticles: Emerging Trends and Future Perspectives. Int. J. Pharm..

[172] Walsh S.E., Maillard J.-Y., Russell A.D., Catrenich C.E., Charbonneau D.L., Bartolo R.G. (2003). Development of Bacterial Resistance to Several Biocides and Effects on Antibiotic Susceptibility. J. Hosp. Infect..

[173] Ferguson A.W. (2003). Comparison of 5% Povidone-Iodine Solution against 1% Povidone-Iodine Solution in Preoperative Cataract Surgery Antisepsis: A Prospective Randomised Double Blind Study. Br. J. Ophthalmol..

[174] Leaper D. (2011). Topical Antiseptics in Wound Care: Time for Reflection. Int. Wound J..

[175] Gupta S., Shinde R.K., Shinde S. (2022). Comparison of the Outcomes of Cadexomer Iodine and Povidone-Iodine Ointments in Wound Management. Cureus.

[176] Kekul Ö., Üstün B., Kömürcü Ö., Bi̇Lgi̇N S., Karakaya D. (2021). Skin Reaction Related to Povidone Iodine Use. JECM.

[177] Eming S.A., Smola-Hess S., Kurschat P., Hirche D., Krieg T., Smola H. (2006). A Novel Property of Povidon-Iodine: Inhibition of Excessive Protease Levels in Chronic Non-Healing Wounds. J. Investig. Dermatol..

[178] Gupta S., Shinde S., Shinde R.K. (2022). Topical Management of Wound: A Narrative Review of Cadexomer Iodine Ointment Versus Povidone Iodine Ointment. Cureus.

[179] Aramwit P., Muangman P., Namviriyachote N., Srichana T. (2010). In Vitro Evaluation of the Antimicrobial Effectiveness and Moisture Binding Properties of Wound Dressings. Int. J. Mol. Sci..

[180] Van Den Poel B., Saegeman V., Schuermans A. (2022). Increasing Usage of Chlorhexidine in Health Care Settings: Blessing or Curse? A Narrative Review of the Risk of Chlorhexidine Resistance and the Implications for Infection Prevention and Control. Eur. J. Clin. Microbiol. Infect. Dis..

[181] Konop M., Damps T., Misicka A., Rudnicka L. (2016). Certain Aspects of Silver and Silver Nanoparticles in Wound Care: A Minireview. J. Nanomater..

[182] Pelgrift R.Y., Friedman A.J. (2013). Nanotechnology as a Therapeutic Tool to Combat Microbial Resistance. Adv. Drug Deliv. Rev..

[183] Hermans M.H. (2006). Silver-Containing Dressings and the Need for Evidence. AJN Am. J. Nurs..

[184] Maillard J.-Y., Hartemann P. (2013). Silver as an Antimicrobial: Facts and Gaps in Knowledge. Crit. Rev. Microbiol..

[185] Peng H., Dong D., Feng S., Guo Y., Yu J., Gan C., Hu X., Qin Z., Liu Y., Gao Y. (2025). Metal-Based Antimicrobial Agents in Wound Dressings: Infection Management and the Challenge of Antibiotic Resistance. Chem. Eng. J..

[186] Dror Y., Ophir C., Freeman A. (2020). Silver–Enzyme Hybrids as Wide-Spectrum Antimicrobial Agents. Innovations and Emerging Technologies in Wound Care.

[187] Zhang W., Hu J., Wu H., Lin X., Cai L. (2025). Stimuli-Responsive Hydrogel Dressing for Wound Healing. APL Mater..

[188] Ehtesabi H., Kalji S.-O., Movsesian L. (2022). Smartphone-Based Wound Dressings: A Mini-Review. Heliyon.

[189] Shu W., Wang Y., Zhang X., Li C., Le H., Chang F. (2021). Functional Hydrogel Dressings for Treatment of Burn Wounds. Front. Bioeng. Biotechnol..

[190] Domínguez-Robles J., Cuartas-Gómez E., Dynes S., Utomo E., Anjani Q.K., Detamornrat U., Donnelly R.F., Moreno-Castellanos N., Larrañeta E. (2023). Poly(caprolactone)/Lignin-Based 3D-Printed Dressings Loaded with a Novel Combination of Bioactive Agents for Wound-Healing Applications. Sustain. Mater. Technol..

[191] Unalan I., Schruefer S., Schubert D.W., Boccaccini A.R. (2023). 3D-Printed Multifunctional Hydrogels with Phytotherapeutic Properties: Development of Essential Oil-Incorporated ALG-XAN Hydrogels for Wound Healing Applications. ACS Biomater. Sci. Eng..

